# Supplementary material for: Traumatic surfing injuries in New Zealand: a descriptive epidemiology study
Source: PeerJ. 2021 Oct 22;9:e12334. doi: 10.7717/peerj.12334 (PMC8544252; doi:10.7717/peerj.12334)
Supplement: Supplemental Information 3 [file peerj-09-12334-s003.docx]

Supplementary file 1: Injury Type and Location of Injuries in Surfers in New Zealand

| Site | Type of Injury | Injury 1, n  (n = 821) | Injury 2, n  (n = 46) | Total, n (%)  (n = 867) |
| --- | --- | --- | --- | --- |
| Head/Face | Skin | 96 | 10 | 106 (50.7) |
|  | Bone | 20 | 1 | 21 (10.0) |
|  | Eye | 22 | 4 | 26 (12.4) |
|  | Ear | 13 | - | 13 (6.2) |
|  | Nervous system | 26 | 4 | 30 (14.4) |
|  | Unknown/Other | 11 | 2 | 13 (6.2) |
|  | Total | 188 | 21 | 209 (100) |
| Neck | Skin | 5 | - | 5 (5) |
|  | Bone | 4 | 1 | 5 (5) |
|  | Joint/Ligament | 25 | 2 | 27 (27) |
|  | Muscle/Tendon | 36 | 5 | 41 (41) |
|  | Nerve | 18 | 1 | 19 (19) |
|  | Unknown/Other | 3 | - | 3 (3) |
|  | Total | 91 | 9 | 100 (100) |
| Shoulder | Skin | 1 | - | 1 (1) |
|  | Bone | 1 | - | 1 (1) |
|  | Joint/Ligament | 37 | - | 37 (38.1) |
|  | Muscle/Tendon | 51 | 1 | 52 (53.6) |
|  | Nerve | 4 | - | 4 (4.1) |
|  | Unknown/Other | 2 | - | 2 (2.1) |
|  | Total | 96 | 1 | 97 (100) |
| Arm | Skin | 11 | - | 11 (23.9) |
|  | Bone | 11 | - | 11 (23.9) |
|  | Joint/Ligament | 11 | - | 11 (23.9) |
|  | Muscle/Tendon | 9 | - | 9 (19.6) |
|  | Nerve | 4 | - | 4 (8.7) |
|  | Unknown/Other | 0 | - | 0 (0) |
|  | Total | 46 | - | 46 (100) |
| Ribs/Sternum | Skin | 4 | - | 4 (8.7) |
|  | Bone | 23 | - | 23 (50) |
|  | Joint/Ligament | 9 | - | 9 (19.6) |
|  | Muscle/Tendon | 8 | - | 8 (17.4) |
|  | Nerve | 1 | - | 1 (2.2) |
|  | Unknown/Other | 1 | - | 1 (2.2) |
|  | Total | 46 | - | 46 (100) |
| Upper back | Skin | 1 | - | 1 (3.7) |
|  | Bone | 2 | - | 2 (7.4) |
|  | Joint/Ligament | 10 | - | 10 (37) |
|  | Muscle/Tendon | 9 | - | 9 (33.3) |
|  | Nerve | 5 | - | 5 (18.5) |
|  | Other | - | - | - |
|  | Total | 27 | - | 27 (27) |
| Lower back | Skin | 5 | - | 5 (5.1) |
|  | Bone | 6 | - | 6 (6.1) |
|  | Joint/Ligament | 26 | 1 | 27 (27.3) |
|  | Muscle/Tendon | 28 | 1 | 29 (29.3) |
|  | Nerve | 23 | 2 | 25 (25.3) |
|  | Other | 7 | - | 7 (7.1) |
|  | Total | 95 | 4 | 99 (100) |
| Hip/Groin | Skin | 4 | - | 4 (6.9) |
|  | Bone | 1 | - | 1 (1.7) |
|  | Joint/Ligament | 16 | - | 16 (27.6) |
|  | Muscle/Tendon | 32 | - | 32 (55.2) |
|  | Nerve | 4 | - | 4 (6.9) |
|  | Other | - | 1 | 1 (1.7) |
|  | Total | 57 | 1 | 58 (100) |
| Knee | Skin | 7 | - | 7 (8.5) |
|  | Bone | 1 | - | 1 (1.2) |
|  | Joint/Ligament | 38 | 4 | 42 (51.2) |
|  | Muscle/Tendon | 19 | 2 | 21 (25.6) |
|  | Nerve | 3 | - | 3 (3.7) |
|  | Other | 8 | - | 8 (9.8) |
|  | Total | 76 | 6 | 82 (100) |
| Ankle/  Lower leg | Skin | 35 | 1 | 36 (35) |
|  | Bone | 14 | 1 | 15 (14.6) |
|  | Joint/Ligament | 26 | 2 | 28 (27.2) |
|  | Muscle/Tendon | 21 | - | 21 (20.4) |
|  | Nerve | 3 | - | 3 (2.9) |
|  | Other | - | - | - |
|  | Total | 99 | 4 | 103 (100) |
