# Supplementary material for: Traumatic surfing injuries in New Zealand: a descriptive epidemiology study
Source: PeerJ. 2021 Oct 22;9:e12334. doi: 10.7717/peerj.12334 (PMC8544252; doi:10.7717/peerj.12334)
Supplement: Supplemental Information 4 [file peerj-09-12334-s004.pdf]

Background Information

Surfing is a popular sport in New Zealand and is rapidly growing worldwide. Research surrounding surfing dates back as far as 1977, however currently there are only a few comprehensive studies on surfing injuries. This questionnaire is important to help understand surfing injuries specifically in New Zealand. Consequently this information will contribute to the foundation for injury prevention research.

Information and Consent Page

Please agree to the information below and give your consent at the bottom of this page.

This questionnaire explores firstly demographics (e.g. age, gender, skill level) and then surfing related injuries that you have experienced in the past 12 months. It may take approximately 5-10 minutes to complete.

The information you provide is purely on a voluntary basis and you may remain anonymous if you wish. We do not anticipate any question to cause discomfort or embarrassment. However if this occurs and/or you no longer wish to continue the questionnaire you may withdraw at any point with no repercussions and data already entered will be disposed of.

If you wish to participate in this questionnaire click "I agree and I am 16 years of age or older". Please note that by clicking "I agree and I am 16 years of age or older" you confirm that you have read this page, understand what is involved and are willing to complete the questionnaire.

If you wish to participate in this questionnaire and you are between 8 and 16 years of age, please click "I agree and I am over 8 years and under 16 years of age " and you will be required to have parental/guardian supervision whilst completing this questionnaire. If you do not wish to participate in the questionnaire, simply click "No I disagree" and you will exit the questionnaire.

All personal data in this study will be treated with complete confidentiality and not made accessible to any person outside of the researchers working on this project. Data that contains identifying information will be stored securely for at least 10 years in accordance with guidelines set out by the Unitec Research Ethics Committee. Data may be presented within an academic forum (e.g. conference presentation or published paper) or to interest groups (e.g. surf clubs or to Māori). It will be presented in such a way that no participant will be able to be identified. Data will not be used for any purpose other than this.

This project has been granted ethical approval from the Unitec Research Ethics Committee (UREC 2015-1032. 22.7.15 - 22.7.16).

This research has been conducted by Debbie Remnant, Masters of Osteopathy student at Unitec Institute of Technology. If you have any queries about this study please contact surfing@unitec.ac.nz. For any concerns or complaints regarding the manner in which this research was conducted alternatively contact the ethics committee at ethics@unitec.ac.nz.

Unitec Research Ethics Committee
c/o Research Office and Postgraduate Centre
Penman House, Building 55, Level 1
Unitec Mt Albert Campus, Gate 4, 139 Carrington Road
Mt Albert, Auckland

* 1. Do you give consent

- ◯ Yes I agree, I am 16 years of age or older and have read the Information and Consent page
- ◯ Yes I agree, I am over 8 years and under 16 years of age
- ◯ No I disagree (or I am under 8 years of age)

Passive Parental Consent:

As you are under 16 years of age please ensure you have a parent/guardian who has read the Information and Consent page and has agreed to supervise you while you complete the questionnaire. You can return to read the Information and Consent page by clicking the "Prev" button. Please ask your parent/guardian to then respond to the question below.

* 2. I am the parent or guardian of the questionnaire participant who is between 8 and 16 years of age. I have read the Information and Consent page and I agree to supervise completion of this questionnaire.

- ◯ I agree
- ◯ No I disagree

Surf Participation

* 3. Please indicate your gender

- Male
- Female

* 4. Please enter your age (use numbers only)

* 5. Are you Natural or Goofy footed? (Natural footed is right back leg. Goofy footed is left back leg)

- Natural footed
- Goofy footed

* 6. Please enter your weight in kilograms (between 15 and 200 kg - use numbers only)

* 7. Please enter your height in centimeters (between 100 and 250 cm - use numbers only)

* 8. Have you lived and surfed in New Zealand for at least 6 of the past 12 months?

- Yes
- No

Surf Participation

* 9. Are you a New Zealand citizen/resident who has been living outside of New Zealand for the majority of the past 12 months?

- ◯ Yes
- ◯ No

* 10. How long are you intending to stay in New Zealand during this visit?

- ◯ More than 6 months (including permanently)
- ◯ 1 - 6 months
- ◯ Less than 1 month

11. Which ethnic group(s) do you belong to?

- ☐ New Zealand European
- ☐ Māori
- ☐ Samoan
- ☐ Cook Island Māori
- ☐ Tongan
- ☐ Niuean
- ☐ Chinese
- ☐ Indian
- ☐ Other (please specify)

* 12. What type of surf board do you predominantly use? (only one option)

- ◯ Short Board
- ◯ Mini Mal
- ◯ Long Board (9 ft plus)
- ◯ Equally Short Board & Mini Mal
- ◯ Equally Short Board & Long Board
- ◯ Equally Mini Mal & Long Board

* 13. How many years have you been surfing?

- Less than 1 year
- 1-5 years
- 6-10 years
- 11-15 years
- 16-20 years
- 21-25 years
- 26-30 years
- 31-35 years
- 36 years plus

* 14. During last SUMMER how many WEEKS (out of 26) did you surf? (December 2014- May 2015)

Surf Participation

* 15. During last SUMMER how many HOURS PER WEEK on average did you surf, of the weeks you did surf? (December 2014 and May 2015)
(between 0.5 and 40hrs, use numbers only)

Surf Participation

* 16. During last WINTER how many WEEKS (out of 26) did you surf? (June and November 2015)

Surf Participation

* 17. During last WINTER how many HOURS PER WEEK on average did you surf, of the weeks you did surf?
(June to November 2015)
(between 0.5 and 40hrs, use numbers only)

* 18. What region do you predominantly surf in? (only one option)

- Northland
- Auckland
- Waikato
- Bay of Plenty
- Gisborne
- Hawkes Bay
- Taranaki
- Manawatu-Wanganui
- Wellington
- Tasman
- Nelson
- Marlborough
- Canterbury
- West Coast
- Otago
- Southland
- Other (please specify)

Surf Participation

* 19. What is your surfing ability? (Select the one that most closely relates to you)

- ◯ Absolute beginner: Not yet able to ride the face of a wave and surfs mostly in the white water.
- ◯ Beginner: Able to ride across the face of the wave and may be developing the skill to generate speed by ‘pumping'.
- ◯ Intermediate: Beginning to perform bottom turns, top turns and cutbacks, and possibly perform these consecutively.
- ◯ Advanced: Able to perform bottom turns, top turns and cutbacks consecutively and occasionally perform advanced manoeuvres such as floaters, re-entries or tube riding.
- ◯ Expert: Able to perform advanced manoeuvres consecutively including floaters, re-entries and tube riding.

* 20. Are you able to complete aerial manoeuvres? (i.e. propel yourself and the board in the air and land back on the water standing on your board)

- ◯ Yes
- ◯ No

* 21. Have you PREVIOUSLY or CURRENTLY taken part in any surfing COMPETITIONS?

- ◯ Yes, I CURRENTLY take part in surfing COMPETITIONS
- ◯ Yes, I have PREVIOUSLY taken part in surfing COMPETITIONS
- ◯ No, never

Surf Participation

* 22. What COMPETITIONS have you PREVIOUSLY taken part in?

- [ ] LOCAL competitions (e.g. board-riders clubs)
- [ ] NATIONAL competitions (e.g. scholastics, grom series, pro series, masters)
- [ ] INTERNATIONAL competitions (e.g. ISA, WQS, WCT)

Surf Participation

* 23. What COMPETITIONS are you CURRENTLY (and previously) taking part in?

- [ ] LOCAL competitions (e.g. board-riders clubs)
- [ ] NATIONAL competitions (e.g. scholastics, grom series, pro series, masters)
- [ ] INTERNATIONAL competitions (e.g. ISA, WQS, WCT)

Upper Body Regions

This section involves questions regarding injuries to the UPPER HALF OF THE BODY including the HEAD & FACE, NECK, SHOULDERS, UPPER BACK, RIBS & STERNUM (chest) and ARMS (elbows, wrists & hands).

* 24. In the past 12 months have you experienced any symptoms (e.g. pain) due to any surfing related injuries in the UPPER HALF OF THE BODY?

(Injuries may have been sudden or traumatic, or they may have occurred gradually over time. Also include previous surfing injuries that are still persisting in the past 12 months.)

◯ Yes

◯ No

Head & Face Injury

* 25. How many HEAD or FACE surfing related injuries have you experienced in the past 12 months?

- ◯ 0
- ◯ 1
- ◯ 2 or more (please specify number of injuries)

Head & Face Injury

This questionnaire will allow you to report your two worst HEAD or FACE surfing related injuries that you experienced in the past 12 months.

* 26. Was the onset of your worst HEAD or FACE injury TRAUMATIC or GRADUAL?

- ◯ TRAUMATIC: There was a specific event or sudden impact that occurred while surfing just prior to any symptoms (i.e. pain).
- ◯ GRADUAL: The symptoms (i.e. pain or discomfort) occurred gradually over time. Not one specific event caused this injury, which is aggravated by surfing. This injury may flare up and down, and be affected by the amount of surfing performed.

Second Head & Face Injury

* 27. Was the onset of your next worst HEAD or FACE injury TRAUMATIC or GRADUAL?

- ◯ TRAUMATIC: There was a specific event or sudden impact that occurred while surfing just prior to any symptoms (i.e. pain).
- ◯ GRADUAL: The symptoms (i.e. pain or discomfort) occurred gradually over time. Not one specific event caused this injury, which is aggravated by surfing. This injury may flare up and down, and be affected by the amount of surfing performed.

Traumatic Injury (head or face)

* 28. What structure was injured in this TRAUMATIC HEAD or FACE injury while surfing in the past 12 months?

- [ ] Skin (e.g. cut, graze, bruise, sting or bite)
- [ ] Bone (e.g. fracture)
- [ ] Ear (e.g. burst/perforated eardrum)
- [ ] Eye (e.g. eye socket or eye ball damage)
- [ ] Nervous system (e.g. concussion or other brain injury)
- [ ] Unknown
- [ ] Other (please specify)

* 29. How long did this TRAUMATIC HEAD or FACE injury take to recover OR is this injury still persisting?

- ○ Less than 3 months to recover
- ○ More than 3 months to recover
- ○ Still persisting and started less than 3 months ago
- ○ Still persisting and started more than 3 months ago

* 30. What was the movement or event that occurred just before or contributed to this TRAUMATIC HEAD or FACE injury?

- Struck by own board
- Struck by other surfer's board
- Striking sea floor
- Striking surface of sea
- Paddling
- Duck diving
- Take off
- Bottom turn
- Top turn
- Cut back
- Re-entry
- Floater
- Aerial
- Riding the face of the wave
- Tube riding
- Encounter with marine life
- Other (please specify)

* 31. What time of year did this TRAUMATIC HEAD or FACE injury occur?

- Summer time (December to May)
- Winter time (June to November)

* 32. What size was the surf when this TRAUMATIC HEAD or FACE injury occurred?

- Overhead or greater in height
- Head height or smaller
- Not sure

* 33. How did you manage this TRAUMATIC HEAD or FACE injury? (you may select more than one option)

- [ ] I had an ACC claim for this injury
- [ ] I went to a healthcare practitioner (e.g. doctor, physiotherapist, osteopath, chiropractor, specialist)
- [ ] I went to a practitioner for traditional Māori healing (e.g. mirimiri, rongoā or karakia)
- [ ] I went to the hospital emergency department
- [ ] I stayed in hospital overnight
- [ ] I had surgery
- [ ] I didn't do anything
- [ ] I managed it myself (e.g. ice, pain killers)
- [ ] Other (please specify, e.g. naturopath, homeopath, acupuncture)

* 34. How much time off SURFING did you take due to this TRAUMATIC HEAD or FACE injury?

- ○ No time off surfing
- ○ More than 1 year off surfing
- ○ Between 1 and 365 days off surfing, please specify number of days (use numbers only)

* 35. How much time off WORK (job or study) did you take due to this TRAUMATIC HEAD or FACE injury?

- ○ No time off work
- ○ More than 1 year off work
- ○ Between 1 and 365 days off work, please specify number of days (use numbers only)

* 36. Have you had another HEAD or FACE injury while surfing in the past 12 months?

- ○ Yes
- ○ No

Traumatic Injury (head or face 2)

* 37. What structure was injured in this TRAUMATIC HEAD or FACE injury while surfing in the past 12 months?

- ☐ Skin (e.g. cut, graze, bruise, sting or bite)
- ☐ Bone (e.g. fracture)
- ☐ Ear (e.g. burst/perforated eardrum)
- ☐ Eye (e.g. eye socket or eye ball damage)
- ☐ Nervous system (e.g. concussion or other brain injury)
- ☐ Unknown
- ☐ Other (please specify)

* 38. How long did this TRAUMATIC HEAD or FACE injury take to recover OR is this injury still persisting?

- ○ Less than 3 months to recover
- ○ More than 3 months to recover
- ○ Still persisting and started less than 3 months ago
- ○ Still persisting and started more than 3 months ago

* 39. What was the movement or event that occurred just before or contributed to this TRAUMATIC HEAD or FACE injury?

- ◯ Struck by own board
- ◯ Struck by other surfers board
- ◯ Striking sea floor
- ◯ Striking surface of sea
- ◯ Paddling
- ◯ Duck diving
- ◯ Take off
- ◯ Bottom turn
- ◯ Top turn
- ◯ Cut back
- ◯ Re-entry
- ◯ Floater
- ◯ Aerial
- ◯ Riding the face of the wave
- ◯ Tube riding
- ◯ Encounter with marine life
- ◯ Other (please specify)

[ ]

* 40. What size was the surf when this TRAUMATIC HEAD or FACE injury occurred?

- ◯ Overhead or greater in height
- ◯ Head height or smaller
- ◯ Not sure

* 41. What time of year did this TRAUMATIC HEAD or FACE injury occur?

- ◯ Summer time (December to May)
- ◯ Winter time (June to November)

* 42. How did you manage this TRAUMATIC HEAD or FACE injury? (you may select more than one option)

- [ ] I had an ACC claim for this injury
- [ ] I went to a healthcare practitioner (e.g. doctor, physiotherapist, osteopath, chiropractor, specialist)
- [ ] I went to a practitioner for traditional Māori healing (e.g. mirimiri, rongoā or karakia)
- [ ] I went to the hospital emergency department
- [ ] I stayed in hospital overnight
- [ ] I had surgery
- [ ] I didn't do anything
- [ ] I managed it myself (e.g. ice, pain killers)
- [ ] Other (please specify, e.g. naturopath, homeopath, acupuncture)

* 43. How much time off SURFING did you take due to this TRAUMATIC HEAD or FACE injury?

- ○ No time off surfing
- ○ More than 1 year off surfing
- ○ Between 1 and 365 days off surfing, please specify number of days (use numbers only)

* 44. How much time off WORK (job or study) did you take due to this TRAUMATIC HEAD or FACE injury?

- ○ No time off work
- ○ More than 1 year off work
- ○ Between 1 and 365 days off work, please specify number of days (use numbers only)

Neck Injury

* 45. How many NECK surfing related injuries have you experienced in the past 12 months?

- 0
- 1
- 2 or more (please specify number of injuries)

Neck Injury

This questionnaire allows you to report your two worst NECK surfing related injuries that you experienced in the past 12 months.

* 46. Was the onset of your worst NECK injury TRAUMATIC or GRADUAL?

- ◯ TRAUMATIC: There was a specific event or sudden impact that occurred while surfing just prior to any symptoms (i.e. pain).
- ◯ GRADUAL: The symptoms (i.e. pain or discomfort) occurred gradually over time. Not one specific event caused this injury, which is aggravated by surfing. This injury may flare up and down, and be affected by the amount of surfing performed.

Second Neck Injury

* 47. Was the onset of your next worst NECK injury TRAUMATIC or GRADUAL?

- ◯ TRAUMATIC: There was a specific event or sudden impact that occurred while surfing just prior to any symptoms (i.e. pain).
- ◯ GRADUAL: The symptoms (i.e. pain or discomfort) occurred gradually over time. Not one specific event caused this injury, which is aggravated by surfing. This injury may flare up and down, and be affected by the amount of surfing performed.

Traumatic Injury (neck)

* 48. What structure was injured in this TRAUMATIC NECK injury while surfing in the past 12 months?

- ☐ Skin (e.g. cut, graze, bruise, bite, sting)
- ☐ Bone (e.g. fracture)
- ☐ Joint or ligament (e.g. sprain, disc or cartilage damage)
- ☐ Muscle and tendon (e.g. tear, strain, rupture)
- ☐ Nerve (e.g. compression causing tingling, numbness etc.)
- ☐ Unknown
- ☐ Other (please specify)

* 49. How long did this TRAUMATIC NECK injury take to recover OR is this injury still persisting?

- ○ Less than 3 months to recover
- ○ More than 3 months to recover
- ○ Still persisting and started less than 3 months ago
- ○ Still persisting and started more than 3 months ago

* 50. What was the movement or event that occurred just before or contributed to this TRAUMATIC NECK injury?

- ◯ Struck by own board
- ◯ Struck by other surfers board
- ◯ Striking sea floor
- ◯ Striking surface of sea
- ◯ Paddling
- ◯ Duck diving
- ◯ Take off
- ◯ Bottom turn
- ◯ Top turn
- ◯ Cut back
- ◯ Re-entry
- ◯ Floater
- ◯ Aerial
- ◯ Riding the face of the wave
- ◯ Tube riding
- ◯ Encounter with marine life
- ◯ Other (please specify)

____

* 51. What size was the surf when this TRAUMATIC NECK injury occurred?

- ◯ Overhead or greater in height
- ◯ Head height or smaller
- ◯ Not sure

* 52. What time of year did this TRAUMATIC NECK injury occur?

- ◯ Summer time (December to May)
- ◯ Winter time (June to November)

* 53. How did you manage this TRAUMATIC NECK injury? (you may select more than one option)

- [ ] I had an ACC claim for this injury
- [ ] I went to a healthcare practitioner (e.g. doctor, physiotherapist, osteopath, chiropractor, specialist)
- [ ] I went to a practitioner for traditional Māori healing (e.g. mirimiri, rongoā or karakia)
- [ ] I went to the hospital emergency department
- [ ] I stayed in hospital over night
- [ ] I had surgery
- [ ] I didn't do anything
- [ ] I managed it myself (e.g. ice, pain killers)
- [ ] Other (please specify, e.g. naturopath, homeopath, acupuncture)

* 54. How much time off SURFING did you take due to this TRAUMATIC NECK injury?

- ○ No time off surfing
- ○ More than 1 year off surfing
- ○ Between 1 and 365 days off surfing, please specify number of days (use numbers only)

* 55. How much time off WORK (job or study) did you take due to this TRAUMATIC NECK injury?

- ○ No time off work
- ○ More than 1 year off work
- ○ Between 1 and 365 days off work, please specify number of days (use numbers only)

* 56. Have you had another NECK injury while surfing in the past 12 months?

- ○ Yes
- ○ No

Traumatic Injury (neck 2)

* 57. What structure was injured in this TRAUMATIC NECK injury while surfing in the past 12 months?

- [ ] Skin (e.g. cut, graze, bruise, bite, sting)
- [ ] Bone (e.g. fracture)
- [ ] Joint or ligament (e.g. sprain, disc or cartilage damage)
- [ ] Muscle and tendon (e.g. tear, strain, rupture)
- [ ] Nerve (e.g. compression causing tingling, numbness etc.)
- [ ] Unknown
- [ ] Other (please specify)

* 58. How long did this TRAUMATIC NECK injury take to recover OR is this injury still persisting?

- ○ Less than 3 months to recover
- ○ More than 3 months to recover
- ○ Still persisting and started less than 3 months ago
- ○ Still persisting and started more than 3 months ago

* 59. What was the movement or event that occurred just before or contributed to this TRAUMATIC NECK injury?

- ◯ Struck by own board
- ◯ Struck by other surfers board
- ◯ Striking sea floor
- ◯ Striking surface of sea
- ◯ Paddling
- ◯ Duck diving
- ◯ Take off
- ◯ Bottom turn
- ◯ Top turn
- ◯ Cut back
- ◯ Re-entry
- ◯ Floater
- ◯ Aerial
- ◯ Riding the face of the wave
- ◯ Tube riding
- ◯ Encounter with marine life
- ◯ Other (please specify)

* 60. What size was the surf when this TRAUMATIC NECK injury occurred?

- ◯ Overhead or greater in height
- ◯ Head height or smaller
- ◯ Not sure

* 61. What time of year did this TRAUMATIC NECK injury occur?

- ◯ Summer time (December to May)
- ◯ Winter time (June to November)

* 62. How did you manage this TRAUMATIC NECK injury? (you may select more than one option)

- [ ] I had an ACC claim for this injury
- [ ] I went to a healthcare practitioner (e.g. doctor, physiotherapist, osteopath, chiropractor, specialist)
- [ ] I went to a practitioner for traditional Māori healing (e.g. mirimiri, rongoā or karakia)
- [ ] I went to the hospital emergency department
- [ ] I stayed in hospital over night
- [ ] I had surgery
- [ ] I didn't do anything
- [ ] I managed it myself (e.g. ice, pain killers)
- [ ] Other (please specify, e.g. naturopath, homeopath, acupuncture)

* 63. How much time off SURFING did you take due to this TRAUMATIC NECK injury?

- ○ No time off surfing
- ○ More than 1 year off surfing
- ○ Between 1 and 365 days off surfing, please specify number of days (use numbers only)

* 64. How much time off WORK (job or study) did you take due to this TRAUMATIC NECK injury?

- ○ No time off work
- ○ More than 1 year off work
- ○ Between 1 and 365 days off work, please specify number of days (use numbers only)

Shoulder Injury

* 65. How many SHOULDER surfing related injuries have you experienced in the past 12 months?

- 0
- 1
- 2 or more (please specify number of injuries)

Shoulder Injury

This questionnaire allows you to report your two worst SHOULDER surfing related injuries that you experienced in the past 12 months.

* 66. Was the onset of your worst SHOULDER injury TRAUMATIC or GRADUAL?

- ◯ TRAUMATIC: There was a specific event or sudden impact that occurred while surfing just prior to any symptoms (i.e. pain).
- ◯ GRADUAL: The symptoms (i.e. pain or discomfort) occurred gradually over time. Not one specific event caused this injury, which is aggravated by surfing. This injury may flare up and down, and be affected by the amount of surfing performed.

Second Shoulder Injury

* 67. Was the onset of your next worst SHOULDER injury TRAUMATIC or GRADUAL?

- ◯ TRAUMATIC: There was a specific event or sudden impact that occurred while surfing just prior to any symptoms (i.e. pain).
- ◯ GRADUAL: The symptoms (i.e. pain or discomfort) occurred gradually over time. Not one specific event caused this injury, which is aggravated by surfing. This injury may flare up and down, and be affected by the amount of surfing performed.

Traumatic Injury (shoulder)

* 68. What structure was injured in this TRAUMATIC SHOULDER injury while surfing in the past 12 months?

- [ ] Skin (e.g. cut, graze, bruise, sting, bite)
- [ ] Bone (e.g. fracture)
- [ ] Joint or ligament (e.g. dislocation, sprain, labrum or cartilage damage)
- [ ] Muscle and tendon (e.g. rotator cuff tear, strain, rupture)
- [ ] Nerve (e.g. compression causing tingling, numbness etc.)
- [ ] Unknown
- [ ] Other (please specify)

* 69. Have you been given a diagnosis for your TRAUMATIC SHOULDER injury from a healthcare practitioner (e.g. doctor, physiotherapist, osteopath, specialist)

- ( ) Yes
- ( ) No

Traumatic Injury (shoulder 2)

* 70. What structure was injured in this TRAUMATIC SHOULDER injury while surfing in the past 12 months?

- [ ] Skin (e.g. cut, graze, bruise, sting, bite)
- [ ] Bone (e.g. fracture)
- [ ] Joint or ligament (e.g. dislocation, sprain, labrum or cartilage damage)
- [ ] Muscle and tendon (e.g. rotator cuff tear, strain, rupture)
- [ ] Nerve (e.g. compression causing tingling, numbness etc.)
- [ ] Unknown
- [ ] Other (please specify)

* 71. Have you been given a diagnosis for your TRAUMATIC SHOULDER injury from a healthcare practitioner (e.g. doctor, physiotherapist, osteopath, specialist)

- ( ) Yes
- ( ) No

Traumatic Injury (shoulder ...)

72. What was the diagnosis of your TRAUMATIC SHOULDER injury?

- ☐ Ligament sprain
- ☐ Dislocation or subluxation (partial dislocation)
- ☐ Cartilage damage
- ☐ Rotator cuff injury
- ☐ AC joint damage
- ☐ Unknown
- ☐ Other (please specify)

* 73. How long did this TRAUMATIC SHOULDER injury take to recover OR is this injury still persisting?

- ○ Less than 3 months to recover
- ○ More than 3 months to recover
- ○ Still persisting and started less than 3 months ago
- ○ Still persisting and started more than 3 months ago

* 74. What was the movement or event that occurred just before or contributed to this TRAUMATIC SHOULDER injury?

- ◯ Struck by own board
- ◯ Struck by other surfers board
- ◯ Striking sea floor
- ◯ Striking surface of sea
- ◯ Paddling
- ◯ Duck diving
- ◯ Take off
- ◯ Bottom turn
- ◯ Top turn
- ◯ Cut back
- ◯ Re-entry
- ◯ Floater
- ◯ Aerial
- ◯ Riding the face of the wave
- ◯ Tube riding
- ◯ Encounter with marine life
- ◯ Other (please specify)

* 75. What size was the surf when this TRAUMATIC SHOULDER injury occurred?

- ◯ Overhead or greater in height
- ◯ Head height or smaller
- ◯ Not sure

* 76. What time of year did this TRAUMATIC SHOULDER injury occur?

- ◯ Summer time (December to May)
- ◯ Winter time (June to November)

* 77. How did you manage this TRAUMATIC SHOULDER injury? (you may select more than one option)

- ☐ I had an ACC claim for this injury
- ☐ I went to a healthcare practitioner (e.g. doctor, physiotherapist, osteopath, chiropractor, specialist)
- ☐ I went to a practitioner for traditional Māori healing (e.g. mirimiri, rongoā or karakia)
- ☐ I went to the hospital emergency department
- ☐ I stayed in hospital over night
- ☐ I had surgery
- ☐ I didn't do anything
- ☐ I managed it myself (e.g. ice, pain killers)
- ☐ Other (please specify, e.g. naturopath, homeopath, acupuncture)

* 78. How much time off SURFING did you take due to this TRAUMATIC SHOULDER injury?

- ○ No time off surfing
- ○ More than 1 year off surfing
- ○ Between 1 and 365 days off surfing, please specify number of days (use numbers only)

* 79. How much time off WORK (job or study) did you take due to this TRAUMATIC SHOULDER injury?

- ○ No time off work
- ○ More than 1 year off work
- ○ Between 1 and 365 days off work, please specify number of days (use numbers only)

* 80. Have you had another SHOULDER injury while surfing in the past 12 months?

- ○ Yes
- ○ No

Traumatic Injury (shoulder ... 2)

81. What was the diagnosis of your TRAUMATIC SHOULDER injury?

- [ ] Ligament sprain
- [ ] Dislocation or subluxation (partial dislocation)
- [ ] Cartilage damage
- [ ] Rotator cuff injury
- [ ] AC joint damage
- [ ] Unknown
- [ ] Other (please specify)

* 82. How long did this TRAUMATIC SHOULDER injury take to recover OR is this injury still persisting?

- ○ Less than 3 months to recover
- ○ More than 3 months to recover
- ○ Still persisting and started less than 3 months ago
- ○ Still persisting and started more than 3 months ago

* 83. What was the movement or event that occurred just before or contributed to this TRAUMATIC SHOULDER injury?

- ○ Struck by own board
- ○ Struck by other surfers board
- ○ Striking sea floor
- ○ Striking surface of sea
- ○ Paddling
- ○ Duck diving
- ○ Take off
- ○ Bottom turn
- ○ Top turn
- ○ Cut back
- ○ Re-entry
- ○ Floater
- ○ Aerial
- ○ Riding the face of the wave
- ○ Tube riding
- ○ Encounter with marine life
- ○ Other (please specify)

* 84. What size was the surf when this TRAUMATIC SHOULDER injury occurred?

- ○ Overhead or greater in height
- ○ Head height or smaller
- ○ Not sure

* 85. What time of year did this TRAUMATIC SHOULDER injury occur?

- ○ Summer time (December to May)
- ○ Winter time (June to November)

* 86. How did you manage this TRAUMATIC SHOULDER injury? (you may select more than one option)

- [ ] I had an ACC claim for this injury
- [ ] I went to a healthcare practitioner (e.g. doctor, physiotherapist, osteopath, chiropractor, specialist)
- [ ] I went to a practitioner for traditional Māori healing (e.g. mirimiri, rongoā or karakia)
- [ ] I went to the hospital emergency department
- [ ] I stayed in hospital over night
- [ ] I had surgery
- [ ] I didn't do anything
- [ ] I managed it myself (e.g. ice, pain killers)
- [ ] Other (please specify, e.g. naturopath, homeopath, acupuncture)

* 87. How much time off SURFING did you take due to this TRAUMATIC SHOULDER injury?

- ○ No time off surfing
- ○ More than 1 year off surfing
- ○ Between 1 and 365 days off surfing, please specify number of days (use numbers only)

* 88. How much time off WORK (job or study) did you take due to this TRAUMATIC SHOULDER injury?

- ○ No time off work
- ○ More than 1 year off work
- ○ Between 1 and 365 days off work, please specify number of days (use numbers only)

Upper Back Injury

* 89. How many UPPER BACK surfing related injuries have you experienced in the past 12 months?

- ○ 0
- ○ 1
- ○ 2 or more (please specify number of injuries)

Upper Back Injury

This questionnaire allows you to report your two worst UPPER BACK surfing related injuries that you experienced in the past 12 months.

* 90. Was the onset of your worst UPPER BACK injury TRAUMATIC or GRADUAL?

- ◯ TRAUMATIC: There was a specific event or sudden impact that occurred while surfing just prior to any symptoms (i.e. pain).
- ◯ GRADUAL: The symptoms (i.e. pain or discomfort) occurred gradually over time. Not one specific event caused this injury, which is aggravated by surfing. This injury may flare up and down, and be affected by the amount of surfing performed.

Second Upper Back Injury

* 91. Was the onset of your next worst UPPER BACK injury TRAUMATIC or GRADUAL?

- ◯ TRAUMATIC: There was a specific event or sudden impact that occurred while surfing just prior to any symptoms (i.e. pain).
- ◯ GRADUAL: The symptoms (i.e. pain or discomfort) occurred gradually over time. Not one specific event caused this injury, which is aggravated by surfing. This injury may flare up and down, and be affected by the amount of surfing performed.

Traumatic Injury (upper back)

* 92. What structure was injured in this TRAUMATIC UPPER BACK injury while surfing in the past 12 months?

- [ ] Skin (e.g. cut, graze, bruise, bite, sting)
- [ ] Bone (e.g. fracture)
- [ ] Joint or ligament (e.g. sprain, disc or cartilage damage)
- [ ] Muscle and tendon (e.g. tear, strain, rupture)
- [ ] Nerve (e.g. compression causing tingling, numbness etc.)
- [ ] Other (please specify)

* 93. How long did this TRAUMATIC UPPER BACK injury take to recover OR is this injury still persisting?

- ○ Less than 3 months to recover
- ○ More than 3 months to recover
- ○ Still persisting and started less than 3 months ago
- ○ Still persisting and started more than 3 months ago

* 94. What was the movement or event that occurred just before or contributed to this TRAUMATIC UPPER BACK injury?

- Struck by own board
- Struck by other surfers board
- Striking sea floor
- Striking surface of sea
- Paddling
- Duck diving
- Take off
- Bottom turn
- Top turn
- Cut back
- Re-entry
- Floater
- Aerial
- Riding the face of the wave
- Tube riding
- Encounter with marine life
- Other (please specify)

* 95. What size was the surf when this TRAUMATIC UPPER BACK injury occurred?

- Overhead or greater in height
- Head height or smaller
- Not sure

* 96. What time of year did this TRAUMATIC UPPER BACK injury occur?

- Summer time (December to May)
- Winter time (June to November)

* 97. How did you manage this TRAUMATIC UPPER BACK injury? (you may select more than one option)

- [ ] I had an ACC claim for this injury
- [ ] I went to a healthcare practitioner (e.g. doctor, physiotherapist, osteopath, chiropractor, specialist)
- [ ] I went to a practitioner for traditional Māori healing (e.g. mirimiri, rongoā or karakia)
- [ ] I went to the hospital emergency department
- [ ] I stayed in hospital over night
- [ ] I had surgery
- [ ] I didn't do anything
- [ ] I managed it myself (e.g. ice, pain killers)
- [ ] Other (please specify, e.g. naturopath, homeopath, acupuncture)

* 98. How much time off SURFING did you take due to this TRAUMATIC UPPER BACK injury?

- ○ No time off surfing
- ○ More than 1 year off surfing
- ○ Between 1 and 365 days off surfing, please specify number of days (use numbers only)

* 99. How much time off WORK (job or study) did you take due to this TRAUMATIC UPPER BACK injury?

- ○ No time off work
- ○ More than 1 year off work
- ○ Between 1 and 365 days off work, please specify number of days (use numbers only)

100. Have you had another UPPER BACK injury while surfing in the past 12 months?

- ○ Yes
- ○ No

Traumatic Injury (upper back 2)

* 101. What structure was injured in this TRAUMATIC UPPER BACK injury while surfing in the past 12 months?

- [ ] Skin (e.g. cut, graze, bruise, bite, sting)
- [ ] Bone (e.g. fracture)
- [ ] Joint or ligament (e.g. sprain, disc or cartilage damage)
- [ ] Muscle and tendon (e.g. tear, strain, rupture)
- [ ] Nerve (e.g. compression causing tingling, numbness etc.)
- [ ] Other (please specify)

* 102. How long did this TRAUMATIC UPPER BACK injury take to recover OR is this injury still persisting?

- ○ Less than 3 months to recover
- ○ More than 3 months to recover
- ○ Still persisting and started less than 3 months ago
- ○ Still persisting and started more than 3 months ago

* 103. What was the movement or event that occurred just before or contributed to this TRAUMATIC UPPER BACK injury?

- ◯ Struck by own board
- ◯ Struck by other surfers board
- ◯ Striking sea floor
- ◯ Striking surface of sea
- ◯ Paddling
- ◯ Duck diving
- ◯ Take off
- ◯ Bottom turn
- ◯ Top turn
- ◯ Cut back
- ◯ Re-entry
- ◯ Floater
- ◯ Aerial
- ◯ Riding the face of the wave
- ◯ Tube riding
- ◯ Encounter with marine life
- ◯ Other (please specify)

* 104. What size was the surf when this TRAUMATIC UPPER BACK injury occurred?

- ◯ Overhead or greater in height
- ◯ Head height or smaller
- ◯ Not sure

* 105. What time of year did this TRAUMATIC UPPER BACK injury occur?

- ◯ Summer time (December to May)
- ◯ Winter time (June to November)

* 106. How did you manage this TRAUMATIC UPPER BACK injury? (you may select more than one option)

- [ ] I had an ACC claim for this injury
- [ ] I went to a healthcare practitioner (e.g. doctor, physiotherapist, osteopath, chiropractor, specialist)
- [ ] I went to a practitioner for traditional Māori healing (e.g. mirimiri, rongoā or karakia)
- [ ] I went to the hospital emergency department
- [ ] I stayed in hospital over night
- [ ] I had surgery
- [ ] I didn't do anything
- [ ] I managed it myself (e.g. ice, pain killers)
- [ ] Other (please specify, e.g. naturopath, homeopath, acupuncture)

* 107. How much time off SURFING did you take due to this TRAUMATIC UPPER BACK injury?

- ○ No time off surfing
- ○ More than 1 year off surfing
- ○ Between 1 and 365 days off surfing, please specify number of days (use numbers only)

* 108. How much time off WORK (job or study) did you take due to this TRAUMATIC UPPER BACK injury?

- ○ No time off work
- ○ More than 1 year off work
- ○ Between 1 and 365 days off work, please specify number of days (use numbers only)

Ribs & Sternum Injury (chest)

* 109. How many RIBS or STERNUM surfing related injuries have you experienced in the past 12 months?

○ 0

○ 1

○ 2 or more (please specify number of injuries)

Ribs & Sternum Injury (chest)

This questionnaire allows you to report your two worst RIB or STERNUM surfing related injuries that you experienced in the past 12 months.

* 110. Was the onset of your worst RIB or STERNUM injury TRAUMATIC or GRADUAL?

- ○ TRAUMATIC: There was a specific event or sudden impact that occurred while surfing just prior to any symptoms (i.e. pain).
- ○ GRADUAL: The symptoms (i.e. pain or discomfort) occurred gradually over time. Not one specific event caused this injury, which is aggravated by surfing. This injury may flare up and down, and be affected by the amount of surfing performed.

Second Ribs & Sternum Injury (chest)

* 111. Was the onset of your next worst RIB or STERNUM injury TRAUMATIC or GRADUAL?

- ◯ TRAUMATIC: There was a specific event or sudden impact that occurred while surfing just prior to any symptoms (i.e. pain).
- ◯ GRADUAL: The symptoms (i.e. pain or discomfort) occurred gradually over time. Not one specific event caused this injury, which is aggravated by surfing. This injury may flare up and down, and be affected by the amount of surfing performed.

Traumatic Injury (ribs or sternum - chest)

* 112. What structure was injured in this TRAUMATIC RIB or STERNUM injury while surfing in the past 12 months?

- [ ] Skin (e.g. cut, graze, bruise, bite, sting)
- [ ] Bone (e.g. fracture)
- [ ] Joint or ligament (e.g. sprain, dislocation, cartilage damage)
- [ ] Muscle and tendon (e.g. tear, strain, rupture)
- [ ] Nerve (e.g. compression causing tingling, numbness etc.)
- [ ] Unknown
- [ ] Other (please specify)

* 113. How long did this TRAUMATIC RIB or STERNUM injury take to recover OR is this injury still persisting?

- ○ Less than 3 months to recover
- ○ More than 3 months to recover
- ○ Still persisting and started less than 3 months ago
- ○ Still persisting and started more than 3 months ago

* 114. What was the movement or event that occurred just before or contributed to this TRAUMATIC RIB or STERNUM injury?

- Struck by own board
- Struck by other surfers board
- Striking sea floor
- Striking surface of sea
- Paddling
- Duck diving
- Take off
- Bottom turn
- Top turn
- Cut back
- Re-entry
- Floater
- Aerial
- Riding the face of the wave
- Tube riding
- Encounter with marine life
- Other (please specify)

* 115. What size was the surf when this TRAUMATIC RIB or STERNUM injury occurred?

- Overhead or greater in height
- Head height or smaller
- Not sure

* 116. What time of year did this TRAUMATIC RIB or STERNUM injury occur?

- Summer time (December to May)
- Winter time (June to November)

* 117. How did you manage this TRAUMATIC RIB or STERNUM injury? (you may select more than one option)

- [ ] I had an ACC claim for this injury
- [ ] I went to a healthcare practitioner (e.g. doctor, physiotherapist, osteopath, chiropractor, specialist)
- [ ] I went to a practitioner for traditional Māori healing (e.g. mirimiri, rongoā or karakia)
- [ ] I went to the hospital emergency department
- [ ] I stayed in hospital over night
- [ ] I had surgery
- [ ] I didn't do anything
- [ ] I managed it myself (e.g. ice, pain killers)
- [ ] Other (please specify, e.g. naturopath, homeopath, acupuncture)

* 118. How much time off SURFING did you take due to this TRAUMATIC RIB or STERNUM injury?

- ( ) No time off surfing
- ( ) More than 1 year off surfing
- ( ) Between 1 and 365 days off surfing, please specify number of days (use numbers only)

* 119. How much time off WORK (job or study) did you take due to this TRAUMATIC RIB or STERNUM injury?

- ( ) No time off work
- ( ) More than 1 year off work
- ( ) Between 1 and 365 days off work, please specify number of days (use numbers only)

120. Have you had another RIB or STERNUM injury while surfing in the past 12 months?

- ( ) Yes
- ( ) No

Traumatic Injury (ribs or sternum - chest 2)

* 121. What structure was injured in this TRAUMATIC RIB or STERNUM injury while surfing in the past 12 months?

- [ ] Skin (e.g. cut, graze, bruise, bite, sting)
- [ ] Bone (e.g. fracture)
- [ ] Joint or ligament (e.g. sprain, dislocation, cartilage damage)
- [ ] Muscle and tendon (e.g. tear, strain, rupture)
- [ ] Nerve (e.g. compression causing tingling, numbness etc.)
- [ ] Unknown
- [ ] Other (please specify)

* 122. How long did this TRAUMATIC RIB or STERNUM injury take to recover OR is this injury still persisting?

- ○ Less than 3 months to recover
- ○ More than 3 months to recover
- ○ Still persisting and started less than 3 months ago
- ○ Still persisting and started more than 3 months ago

* 123. What was the movement or event that occurred just before or contributed to this TRAUMATIC RIB or STERNUM injury?

- Struck by own board
- Struck by other surfers board
- Striking sea floor
- Striking surface of sea
- Paddling
- Duck diving
- Take off
- Bottom turn
- Top turn
- Cut back
- Re-entry
- Floater
- Aerial
- Riding the face of the wave
- Tube riding
- Encounter with marine life
- Other (please specify)

* 124. What size was the surf when this TRAUMATIC RIB or STERNUM injury occurred?

- Overhead or greater in height
- Head height or smaller
- Not sure

* 125. What time of year did this TRAUMATIC RIB or STERNUM injury occur?

- Summer time (December to May)
- Winter time (June to November)

* 126. How did you manage this TRAUMATIC RIB or STERNUM injury? (you may select more than one option)

- [ ] I had an ACC claim for this injury
- [ ] I went to a healthcare practitioner (e.g. doctor, physiotherapist, osteopath, chiropractor, specialist)
- [ ] I went to a practitioner for traditional Māori healing (e.g. mirimiri, rongoā or karakia)
- [ ] I went to the hospital emergency department
- [ ] I stayed in hospital over night
- [ ] I had surgery
- [ ] I didn't do anything
- [ ] I managed it myself (e.g. ice, pain killers)
- [ ] Other (please specify, e.g. naturopath, homeopath, acupuncture)

* 127. How much time off SURFING did you take due to this TRAUMATIC RIB or STERNUM injury?

- ( ) No time off surfing
- ( ) More than 1 year off surfing
- ( ) Between 1 and 365 days off surfing, please specify number of days (use numbers only)

* 128. How much time off WORK (job or study) did you take due to this TRAUMATIC RIB or STERNUM injury?

- ( ) No time off work
- ( ) More than 1 year off work
- ( ) Between 1 and 365 days off work, please specify number of days (use numbers only)

Arm Injury (including elbow, wrist & hand)

* 129. How many ARM surfing related injuries (including elbow, wrist and hand) have you experienced in the past 12 months?

- 0
- 1
- 2 or more (please specify number of injuries)

Arm Injury (including elbow, wrist & hand)

This questionnaire allows you to report your two worst ARM (including elbow, wrist & hand) surfing related injuries that you experienced in the past 12 months.

* 130. Was the onset of your worst ARM injury TRAUMATIC or GRADUAL?

- ◯ TRAUMATIC: There was a specific event or sudden impact that occurred while surfing just prior to any symptoms (i.e. pain).
- ◯ GRADUAL: The symptoms (i.e. pain or discomfort) occurred gradually over time. Not one specific event caused this injury, which is aggravated by surfing. This injury may flare up and down, and be affected by the amount of surfing performed.

Second Arm Injury (including elbow, wrist & hand)

* 131. Was the onset of your next worst ARM injury TRAUMATIC or GRADUAL?

- ◯ TRAUMATIC: There was a specific event or sudden impact that occurred while surfing just prior to any symptoms (i.e. pain).
- ◯ GRADUAL: The symptoms (i.e. pain or discomfort) occurred gradually over time. Not one specific event caused this injury, which is aggravated by surfing. This injury may flare up and down, and be affected by the amount of surfing performed.

Traumatic Injury (arm - including elbow, wrist & hand)

* 132. What structure was injured in this TRAUMATIC ARM (elbow, wrist or hand) injury while surfing in the past 12 months?

- [ ] Skin (e.g. cut, graze, bruise, bite, sting)
- [ ] Bone (e.g. fracture)
- [ ] Joint or ligament (e.g. sprain, dislocation, disc or cartilage damage)
- [ ] Muscle and tendon (e.g. tear, strain, rupture)
- [ ] Nerve (e.g. compression causing tingling, numbness etc.)
- [ ] Unknown
- [ ] Other (please specify)

* 133. How long did this TRAUMATIC ARM injury take to recover OR is this injury still persisting?

- ○ Less than 3 months to recover
- ○ More than 3 months to recover
- ○ Still persisting and started less than 3 months ago
- ○ Still persisting and started more than 3 months ago

* 134. What was the movement or event that occurred just before or contributed to this TRAUMATIC ARM injury?

- ◯ Struck by own board
- ◯ Struck by other surfers board
- ◯ Striking sea floor
- ◯ Striking surface of sea
- ◯ Paddling
- ◯ Duck diving
- ◯ Take off
- ◯ Bottom turn
- ◯ Top turn
- ◯ Cut back
- ◯ Re-entry
- ◯ Floater
- ◯ Aerial
- ◯ Riding the face of the wave
- ◯ Tube riding
- ◯ Encounter with marine life
- ◯ Other (please specify)

* 135. What size was the surf when this TRAUMATIC ARM injury occurred?

- ◯ Overhead or greater in height
- ◯ Head height or smaller
- ◯ Not sure

* 136. What time of year did this TRAUMATIC ARM injury occur?

- ◯ Summer time (December to May)
- ◯ Winter time (June to November)

* 137. How did you manage this TRAUMATIC ARM injury? (you may select more than one option)

- [ ] I had an ACC claim for this injury
- [ ] I went to a healthcare practitioner (e.g. doctor, physiotherapist, osteopath, chiropractor, specialist)
- [ ] I went to a practitioner for traditional Māori healing (e.g. mirimiri, rongoā or karakia)
- [ ] I went to the hospital emergency department
- [ ] I stayed in hospital over night
- [ ] I had surgery
- [ ] I didn't do anything
- [ ] I managed it myself (e.g. ice, pain killers)
- [ ] Other (please specify, e.g. naturopath, homeopath, acupuncture)

* 138. How much time off SURFING did you take due to this TRAUMATIC ARM injury?

- ○ No time off surfing
- ○ More than 1 year off surfing
- ○ Between 1 and 365 days off surfing, please specify number of days (use numbers only)

* 139. How much time off WORK (job or study) did you take due to this TRAUMATIC ARM injury?

- ○ No time off work
- ○ More than 1 year off work
- ○ Between 1 and 365 days off work, please specify number of days (use numbers only)

* 140. Have you had another ARM (elbow, wrist or hand) injury while surfing in the past 12 months?

- ○ Yes
- ○ No

Traumatic Injury (arm - including elbow, wrist & hand 2)

* 141. What structure was injured in this TRAUMATIC ARM (elbow, wrist or hand) injury while surfing in the past 12 months?

- ☐ Skin (e.g. cut, graze, bruise, bite, sting)
- ☐ Bone (e.g. fracture)
- ☐ Joint or ligament (e.g. sprain, dislocation, disc or cartilage damage)
- ☐ Muscle and tendon (e.g. tear, strain, rupture)
- ☐ Nerve (e.g. compression causing tingling, numbness etc.)
- ☐ Unknown
- ☐ Other (please specify)

* 142. How long did this TRAUMATIC ARM injury take to recover OR is this injury still persisting?

- ○ Less than 3 months to recover
- ○ More than 3 months to recover
- ○ Still persisting and started less than 3 months ago
- ○ Still persisting and started more than 3 months ago

* 143. What was the movement or event that occurred just before or contributed to this TRAUMATIC ARM injury?

- ◯ Struck by own board
- ◯ Struck by other surfers board
- ◯ Striking sea floor
- ◯ Striking surface of sea
- ◯ Paddling
- ◯ Duck diving
- ◯ Take off
- ◯ Bottom turn
- ◯ Top turn
- ◯ Cut back
- ◯ Re-entry
- ◯ Floater
- ◯ Aerial
- ◯ Riding the face of the wave
- ◯ Tube riding
- ◯ Encounter with marine life
- ◯ Other (please specify)

* 144. What size was the surf when this TRAUMATIC ARM injury occurred?

- ◯ Overhead or greater in height
- ◯ Head height or smaller
- ◯ Not sure

* 145. What time of year did this TRAUMATIC ARM injury occur?

- ◯ Summer time (December to May)
- ◯ Winter time (June to November)

* 146. How did you manage this TRAUMATIC ARM injury? (you may select more than one option)

- [ ] I had an ACC claim for this injury
- [ ] I went to a healthcare practitioner (e.g. doctor, physiotherapist, osteopath, chiropractor, specialist)
- [ ] I went to a practitioner for traditional Māori healing (e.g. mirimiri, rongoā or karakia)
- [ ] I went to the hospital emergency department
- [ ] I stayed in hospital over night
- [ ] I had surgery
- [ ] I didn't do anything
- [ ] I managed it myself (e.g. ice, pain killers)
- [ ] Other (please specify, e.g. naturopath, homeopath, acupuncture)

* 147. How much time off SURFING did you take due to this TRAUMATIC ARM injury?

- ○ No time off surfing
- ○ More than 1 year off surfing
- ○ Between 1 and 365 days off surfing, please specify number of days (use numbers only)

* 148. How much time off WORK (job or study) did you take due to this TRAUMATIC ARM injury?

- ○ No time off work
- ○ More than 1 year off work
- ○ Between 1 and 365 days off work, please specify number of days (use numbers only)

Lower Body Regions

This section involves questions regarding injuries to the LOWER HALF OF THE BODY including the LOWER BACK (pelvis, sacrum), HIP & GROIN, KNEE and LOWER LEG & ANKLE (foot).

* 149. In the past 12 months have you experienced any symptoms (e.g. pain) due to any surfing related injuries in the LOWER HALF OF THE BODY?

(Injuries may have been sudden or traumatic, or they may have occurred gradually over time. Also include previous surfing injuries that are still persisting in the past 12 months.)

- ◯ Yes
- ◯ No

Lower Back Injury

* 150. How many LOWER BACK surfing related injuries have you experienced in the past 12 months?

- 0
- 1
- 2 or more (please specify number of injuries)

Lower Back Injury

This questionnaire allows you to report your two worst LOWER BACK surfing related injuries that you experienced in the past 12 months.

* 151. Was the onset of your worst LOWER BACK injury TRAUMATIC or GRADUAL?

- ◯ TRAUMATIC: There was a specific event or sudden impact that occurred while surfing just prior to any symptoms (i.e. pain).
- ◯ GRADUAL: The symptoms (i.e. pain or discomfort) occurred gradually over time. Not one specific event caused this injury, which is aggravated by surfing. This injury may flare up and down, and be affected by the amount of surfing performed.

Second Lower Back Injury

* 152. Was the onset of your next worst LOWER BACK injury TRAUMATIC or GRADUAL?

- TRAUMATIC: There was a specific event or sudden impact that occurred while surfing just prior to any symptoms (i.e. pain).
- GRADUAL: The symptoms (i.e. pain or discomfort) occurred gradually over time. Not one specific event caused this injury, which is aggravated by surfing. This injury may flare up and down, and be affected by the amount of surfing performed.

Traumatic Injury (lower back)

* 153. What structure was injured in this TRAUMATIC LOWER BACK injury while surfing in the past 12 months?

- [ ] Skin (e.g. cut, graze, bruise, bite, sting)
- [ ] Bone (e.g. fracture)
- [ ] Joint or ligament (e.g. sprain, disc or cartilage damage)
- [ ] Muscle and tendon (e.g. tear, strain, rupture)
- [ ] Nerve (e.g.compression causing tingling, numbness etc.)
- [ ] Unknown
- [ ] Other (please specify)

* 154. How long did this TRAUMATIC LOWER BACK injury take to recover OR is this injury still persisting?

- ○ Less than 3 months to recover
- ○ More than 3 months to recover
- ○ Still persisting and started less than 3 months ago
- ○ Still persisting and started more than 3 months ago

* 155. What was the movement or event that occurred just before or contributed to this TRAUMATIC LOWER BACK injury?

- ◯ Struck by own board
- ◯ Struck by other surfers board
- ◯ Striking sea floor
- ◯ Striking surface of sea
- ◯ Paddling
- ◯ Duck diving
- ◯ Take off
- ◯ Bottom turn
- ◯ Top turn
- ◯ Cut back
- ◯ Re-entry
- ◯ Floater
- ◯ Aerial
- ◯ Riding the face of the wave
- ◯ Tube riding
- ◯ Encounter with marine life
- ◯ Other (please specify)

* 156. What size was the surf when this TRAUMATIC LOWER BACK injury occurred?

- ◯ Overhead or greater in height
- ◯ Head height or smaller
- ◯ Not sure

* 157. What time of year did this TRAUMATIC LOWER BACK injury occur?

- ◯ Summer time (December to May)
- ◯ Winter time (June to November)

* 158. How did you manage this TRAUMATIC LOWER BACK injury? (you may select more than one option)

- [ ] I had an ACC claim for this injury
- [ ] I went to a healthcare practitioner (e.g. doctor, physiotherapist, osteopath, chiropractor, specialist)
- [ ] I went to a practitioner for traditional Māori healing (e.g. mirimiri, rongoā or karakia)
- [ ] I went to the hospital emergency department
- [ ] I stayed in hospital over night
- [ ] I had surgery
- [ ] I didn't do anything
- [ ] I managed (e.g. ice, pain killers)
- [ ] Other (please specify, e.g. naturopath, homeopath, acupuncture)

* 159. How much time off SURFING did you take due to this TRAUMATIC LOWER BACK injury?

- ○ No time off surfing
- ○ More than 1 year off surfing
- ○ Between 1 and 365 days off surfing, please specify number of days (use numbers only)

* 160. How much time off WORK (job or study) did you take due to this TRAUMATIC LOWER BACK injury?

- ○ No time off work
- ○ More than 1 year off work
- ○ Between 1 and 365 days off work, please specify number of days (use numbers only)

* 161. Have you had another LOWER BACK injury while surfing in the past 12 months?

- ○ Yes
- ○ No

Traumatic Injury (lower back 2)

* 162. What structure was injured in this TRAUMATIC LOWER BACK injury while surfing in the past 12 months?

- [ ] Skin (e.g. cut, graze, bruise, bite, sting)
- [ ] Bone (e.g. fracture)
- [ ] Joint or ligament (e.g. sprain, disc or cartilage damage)
- [ ] Muscle and tendon (e.g. tear, strain, rupture)
- [ ] Nerve (e.g.compression causing tingling, numbness etc.)
- [ ] Unknown
- [ ] Other (please specify)

* 163. How long did this TRAUMATIC LOWER BACK injury take to recover OR is this injury still persisting?

- ○ Less than 3 months to recover
- ○ More than 3 months to recover
- ○ Still persisting and started less than 3 months ago
- ○ Still persisting and started more than 3 months ago

* 164. What was the movement or event that occurred just before or contributed to this TRAUMATIC LOWER BACK injury?

- ◯ Struck by own board
- ◯ Struck by other surfers board
- ◯ Striking sea floor
- ◯ Striking surface of sea
- ◯ Paddling
- ◯ Duck diving
- ◯ Take off
- ◯ Bottom turn
- ◯ Top turn
- ◯ Cut back
- ◯ Re-entry
- ◯ Floater
- ◯ Aerial
- ◯ Riding the face of the wave
- ◯ Tube riding
- ◯ Encounter with marine life
- ◯ Other (please specify)

* 165. What size was the surf when this TRAUMATIC LOWER BACK injury occurred?

- ◯ Overhead or greater in height
- ◯ Head height or smaller
- ◯ Not sure

* 166. What time of year did this TRAUMATIC LOWER BACK injury occur?

- ◯ Summer time (December to May)
- ◯ Winter time (June to November)

* 167. How did you manage this TRAUMATIC LOWER BACK injury? (you may select more than one option)

- [ ] I had an ACC claim for this injury
- [ ] I went to a healthcare practitioner (e.g. doctor, physiotherapist, osteopath, chiropractor, specialist)
- [ ] I went to a practitioner for traditional Māori healing (e.g. mirimiri, rongoā or karakia)
- [ ] I went to the hospital emergency department
- [ ] I stayed in hospital over night
- [ ] I had surgery
- [ ] I didn't do anything
- [ ] I managed (e.g. ice, pain killers)
- [ ] Other (please specify, e.g. naturopath, homeopath, acupuncture)

* 168. How much time off SURFING did you take due to this TRAUMATIC LOWER BACK injury?

- ( ) No time off surfing
- ( ) More than 1 year off surfing
- ( ) Between 1 and 365 days off surfing, please specify number of days (use numbers only)

* 169. How much time off WORK (job or study) did you take due to this TRAUMATIC LOWER BACK injury?

- ( ) No time off work
- ( ) More than 1 year off work
- ( ) Between 1 and 365 days off work, please specify number of days (use numbers only)

Hip & Groin Injury

* 170. How many HIP or GROIN surfing related injuries have you experienced in the past 12 months?

- ◯ 0
- ◯ 1
- ◯ 2 or more (please specify number of injuries)

Hip & Groin Injury

This questionnaire allows you to report your two worst HIP or GROIN surfing related injuries that you experienced in the past 12 months.

* 171. Was the onset of your worst HIP or GROIN injury TRAUMATIC or GRADUAL?

◯ TRAUMATIC: There was a specific event or sudden impact that occurred while surfing just prior to any symptoms (i.e. pain).

◯ GRADUAL: The symptoms (i.e. pain or discomfort) occurred gradually over time. Not one specific event caused this injury, which is aggravated by surfing. This injury may flare up and down, and be affected by the amount of surfing performed.

Second Hip & Groin Injury

* 172. Was the onset of your next worst HIP or GROIN injury TRAUMATIC or GRADUAL?

- ◯ TRAUMATIC: There was a specific event or sudden impact that occurred while surfing just prior to any symptoms (i.e. pain).
- ◯ GRADUAL: The symptoms (i.e. pain or discomfort) occurred gradually over time. Not one specific event caused this injury, which is aggravated by surfing. This injury may flare up and down, and be affected by the amount of surfing performed.

Traumatic Injury (hip & groin)

* 173. Was this TRAUMATIC HIP or GROIN injury on your front or back leg

- ◯ Front leg
- ◯ Back leg

* 174. What structure was injured in this TRAUMATIC HIP or GROIN injury while surfing in the past 12 months?

- ☐ Skin (e.g. cut, graze, bruise, bite, sting)
- ☐ Bone (e.g. fracture)
- ☐ Joint or ligament (e.g. sprain, labrum or cartilage damage)
- ☐ Muscle and tendon (e.g. tear, strain, rupture)
- ☐ Nerve (e.g. compression causing tingling, numbness etc.)
- ☐ Unknown
- ☐ Other (please specify)

* 175. How long did this TRAUMATIC HIP or GROIN injury take to recover OR is this injury still persisting?

- ◯ Less than 3 months to recover
- ◯ More than 3 months to recover
- ◯ Still persisting and started less than 3 months ago
- ◯ Still persisting and started more than 3 months ago

* 176. What was the movement or event that occurred just before or contributed to this TRAUMATIC HIP or GROIN injury?

- ◯ Struck by own board
- ◯ Struck by other surfers board
- ◯ Striking sea floor
- ◯ Striking surface of sea
- ◯ Paddling
- ◯ Duck diving
- ◯ Take off
- ◯ Bottom turn
- ◯ Top turn
- ◯ Cut back
- ◯ Re-entry
- ◯ Floater
- ◯ Aerial
- ◯ Riding the face of the wave
- ◯ Tube riding
- ◯ Encounter with marine life
- ◯ Other (please specify)

* 177. What size was the surf when this TRAUMATIC HIP or GROIN injury occurred?

- ◯ Overhead or greater in height
- ◯ Head height or smaller
- ◯ Not sure

* 178. What time of year did this TRAUMATIC HIP or GROIN injury occur?

- ◯ Summer time (December to May)
- ◯ Winter time (June to November)

* 179. How did you manage this TRAUMATIC HIP or GROIN injury? (you may select more than one option)

- [ ] I had an ACC claim for this injury
- [ ] I went to a healthcare practitioner (e.g. doctor, physiotherapist, osteopath, chiropractor, specialist)
- [ ] I went to a practitioner for traditional Māori healing (e.g. mirimiri, rongoā or karakia)
- [ ] I went to the hospital emergency department
- [ ] I stayed in hospital over night
- [ ] I had surgery
- [ ] I didn't do anything
- [ ] I managed it myself (e.g. ice, pain killers)
- [ ] Other (please specify, e.g. naturopath, homeopath, acupuncture)

* 180. How much time off SURFING did you take due to this TRAUMATIC HIP or GROIN injury?

- ( ) No time off surfing
- ( ) More than 1 year off surfing
- ( ) Between 1 and 365 days off surfing, please specify number of days (use numbers only)

* 181. How much time off WORK (job or study) did you take due to this TRAUMATIC HIP or GROIN injury?

- ( ) No time off work
- ( ) More than 1 year off work
- ( ) Between 1 and 365 days off work, please specify number of days (use numbers only)

* 182. Have you had another HIP or GROIN injury while surfing in the past 12 months?

- ( ) Yes
- ( ) No

Traumatic Injury (hip & groin 2)

* 183. Was this TRAUMATIC HIP or GROIN injury on your front or back leg

- ○ Front leg
- ○ Back leg

* 184. What structure was injured in this TRAUMATIC HIP or GROIN injury while surfing in the past 12 months?

- ☐ Skin (e.g. cut, graze, bruise, bite, sting)
- ☐ Bone (e.g. fracture)
- ☐ Joint or ligament (e.g. sprain, labrum or cartilage damage)
- ☐ Muscle and tendon (e.g. tear, strain, rupture)
- ☐ Nerve (e.g. compression causing tingling, numbness etc.)
- ☐ Unknown
- ☐ Other (please specify)

* 185. How long did this TRAUMATIC HIP or GROIN injury take to recover OR is this injury still persisting?

- ○ Less than 3 months to recover
- ○ More than 3 months to recover
- ○ Still persisting and started less than 3 months ago
- ○ Still persisting and started more than 3 months ago

* 186. What was the movement or event that occurred just before or contributed to this TRAUMATIC HIP or GROIN injury?

- ◯ Struck by own board
- ◯ Struck by other surfers board
- ◯ Striking sea floor
- ◯ Striking surface of sea
- ◯ Paddling
- ◯ Duck diving
- ◯ Take off
- ◯ Bottom turn
- ◯ Top turn
- ◯ Cut back
- ◯ Re-entry
- ◯ Floater
- ◯ Aerial
- ◯ Riding the face of the wave
- ◯ Tube riding
- ◯ Encounter with marine life
- ◯ Other (please specify)

* 187. What size was the surf when this TRAUMATIC HIP or GROIN injury occurred?

- ◯ Overhead or greater in height
- ◯ Head height or smaller
- ◯ Not sure

* 188. What time of year did this TRAUMATIC HIP or GROIN injury occur?

- ◯ Summer time (December to May)
- ◯ Winter time (June to November)

* 189. How did you manage this TRAUMATIC HIP or GROIN injury? (you may select more than one option)

- [ ] I had an ACC claim for this injury
- [ ] I went to a healthcare practitioner (e.g. doctor, physiotherapist, osteopath, chiropractor, specialist)
- [ ] I went to a practitioner for traditional Māori healing (e.g. mirimiri, rongoā or karakia)
- [ ] I went to the hospital emergency department
- [ ] I stayed in hospital over night
- [ ] I had surgery
- [ ] I didn't do anything
- [ ] I managed it myself (e.g. ice, pain killers)
- [ ] Other (please specify, e.g. naturopath, homeopath, acupuncture)

* 190. How much time off SURFING did you take due to this TRAUMATIC HIP or GROIN injury?

- ( ) No time off surfing
- ( ) More than 1 year off surfing
- ( ) Between 1 and 365 days off surfing, please specify number of days (use numbers only)

* 191. How much time off WORK (job or study) did you take due to this TRAUMATIC HIP or GROIN injury?

- ( ) No time off work
- ( ) More than 1 year off work
- ( ) Between 1 and 365 days off work, please specify number of days (use numbers only)

Knee Injury

* 192. How many KNEE surfing related injuries have you experienced in the past 12 months?

- 0
- 1
- 2 or more (please specify number of injuries)

Knee Injury

This questionnaire allows you to report your two worst KNEE surfing related injuries that you experienced in the past 12 months.

* 193. Was the onset of your worst KNEE injury TRAUMATIC or GRADUAL?

- ◯ TRAUMATIC: There was a specific event or sudden impact that occurred while surfing just prior to any symptoms (i.e. pain).
- ◯ GRADUAL: The symptoms (i.e. pain or discomfort) occurred gradually over time. Not one specific event caused this injury, which is aggravated by surfing. This injury may flare up and down, and be affected by the amount of surfing performed.

Second Knee Injury

* 194. Was the onset of your next worst KNEE injury TRAUMATIC or GRADUAL?

- ◯ TRAUMATIC: There was a specific event or sudden impact that occurred while surfing just prior to any symptoms (i.e. pain).
- ◯ GRADUAL: The symptoms (i.e. pain or discomfort) occurred gradually over time. Not one specific event caused this injury, which is aggravated by surfing. This injury may flare up and down, and be affected by the amount of surfing performed.

Traumatic Injury (knee)

* 195. Was this TRAUMATIC KNEE injury on your front or back leg

- ◯ Front leg
- ◯ Back leg

* 196. What structure was injured in this TRAUMATIC KNEE injury while surfing in the past 12 months?

- ☐ Skin (e.g. cut, graze, bruise, bite, sting)
- ☐ Bone (e.g. fracture)
- ☐ Joint or ligament (e.g. sprain, cartilage/meniscus damage, dislocation)
- ☐ Muscle and tendon (e.g. tear, strain, rupture)
- ☐ Nerve (e.g. compression causing tingling, numbness etc.)
- ☐ Unknown
- ☐ Other (please specify)

* 197. Have you been given a diagnosis for your TRAUMATIC KNEE injury from a healthcare practitioner (e.g. doctor, physiotherapist, osteopath, specialist)

- ◯ Yes
- ◯ No

Traumatic Injury (knee 2)

* 198. Was this TRAUMATIC KNEE injury on your front or back leg

- ○ Front leg
- ○ Back leg

* 199. What structure was injured in this TRAUMATIC KNEE injury while surfing in the past 12 months?

- ☐ Skin (e.g. cut, graze, bruise, bite, sting)
- ☐ Bone (e.g. fracture)
- ☐ Joint or ligament (e.g. sprain, cartilage/meniscus damage, dislocation)
- ☐ Muscle and tendon (e.g. tear, strain, rupture)
- ☐ Nerve (e.g. compression causing tingling, numbness etc.)
- ☐ Unknown
- ☐ Other (please specify)

* 200. Have you been given a diagnosis for your TRAUMATIC KNEE injury from a healthcare practitioner (e.g. doctor, physiotherapist, osteopath, specialist)

- ○ Yes
- ○ No

Traumatic Injury (knee...)

201. What was the diagnosis of your TRAUMATIC KNEE injury?

- [ ] Ligament sprain (e.g. ACL or MCL)
- [ ] Cartilage damage/tear (meniscus)
- [ ] Dislocation
- [ ] Unknown
- [ ] Other (please specify)

* 202. How long did this TRAUMATIC KNEE injury take to recover OR is this injury still persisting?

- ○ Less than 3 months to recover
- ○ More than 3 months to recover
- ○ Still persisting and started less than 3 months ago
- ○ Still persisting and started more than 3 months ago

* 203. What was the movement or event that occurred just before or contributed to this TRAUMATIC KNEE injury?

- ◯ Struck by own board
- ◯ Struck by other surfers board
- ◯ Striking sea floor
- ◯ Striking surface of sea
- ◯ Paddling
- ◯ Duck diving
- ◯ Take off
- ◯ Bottom turn
- ◯ Top turn
- ◯ Cut back
- ◯ Re-entry
- ◯ Floater
- ◯ Aerial
- ◯ Riding the face of the wave
- ◯ Tube riding
- ◯ Encounter with marine life
- ◯ Other (please specify)

* 204. What size was the surf when this TRAUMATIC KNEE injury occurred?

- ◯ Overhead or greater in height
- ◯ Head height or smaller
- ◯ Not sure

* 205. What time of year did this TRAUMATIC KNEE injury occur?

- ◯ Summer time (December to May)
- ◯ Winter time (June to November)

* 206. How did you manage this TRAUMATIC KNEE injury? (you may select more than one option)

- [ ] I had an ACC claim for this injury
- [ ] I went to a healthcare practitioner (e.g. doctor, physiotherapist, osteopath, chiropractor, specialist)
- [ ] I went to a practitioner for traditional Māori healing (e.g. mirimiri, rongoā or karakia)
- [ ] I went to the hospital emergency department
- [ ] I stayed in hospital over night
- [ ] I had surgery
- [ ] I didn't do anything
- [ ] I managed it myself (e.g. ice, pain killers)
- [ ] Other (please specify, e.g. naturopath, homeopath, acupuncture)

* 207. How much time off SURFING did you take due to this TRAUMATIC KNEE injury?

- ( ) No time off surfing
- ( ) More than 1 year off surfing
- ( ) Between 1 and 365 days off surfing, please specify number of days (use numbers only)

* 208. How much time off WORK (job or study) did you take due to this TRAUMATIC KNEE injury?

- ( ) No time off work
- ( ) More than 1 year off work
- ( ) Between 1 and 365 days off work, please specify number of days (use numbers only)

* 209. Have you had another KNEE injury while surfing in the past 12 months?

- ( ) Yes
- ( ) No

Traumatic Injury (knee... 2)

210. What was the diagnosis of your TRAUMATIC KNEE injury?

- [ ] Ligament sprain (e.g. ACL or MCL)
- [ ] Cartilage damage/tear (meniscus)
- [ ] Dislocation
- [ ] Unknown
- [ ] Other (please specify)

* 211. How long did this TRAUMATIC KNEE injury take to recover OR is this injury still persisting?

- ○ Less than 3 months to recover
- ○ More than 3 months to recover
- ○ Still persisting and started less than 3 months ago
- ○ Still persisting and started more than 3 months ago

* 212. What was the movement or event that occurred just before or contributed to this TRAUMATIC KNEE injury?

- ◯ Struck by own board
- ◯ Struck by other surfers board
- ◯ Striking sea floor
- ◯ Striking surface of sea
- ◯ Paddling
- ◯ Duck diving
- ◯ Take off
- ◯ Bottom turn
- ◯ Top turn
- ◯ Cut back
- ◯ Re-entry
- ◯ Floater
- ◯ Aerial
- ◯ Riding the face of the wave
- ◯ Tube riding
- ◯ Encounter with marine life
- ◯ Other (please specify)

* 213. What size was the surf when this TRAUMATIC KNEE injury occurred?

- ◯ Overhead or greater in height
- ◯ Head height or smaller
- ◯ Not sure

* 214. What time of year did this TRAUMATIC KNEE injury occur?

- ◯ Summer time (December to May)
- ◯ Winter time (June to November)

* 215. How did you manage this TRAUMATIC KNEE injury? (you may select more than one option)

- [ ] I had an ACC claim for this injury
- [ ] I went to a healthcare practitioner (e.g. doctor, physiotherapist, osteopath, chiropractor, specialist)
- [ ] I went to a practitioner for traditional Māori healing (e.g. mirimiri, rongoā or karakia)
- [ ] I went to the hospital emergency department
- [ ] I stayed in hospital over night
- [ ] I had surgery
- [ ] I didn't do anything
- [ ] I managed it myself (e.g. ice, pain killers)
- [ ] Other (please specify, e.g. naturopath, homeopath, acupuncture)

* 216. How much time off SURFING did you take due to this TRAUMATIC KNEE injury?

- ○ No time off surfing
- ○ More than 1 year off surfing
- ○ Between 1 and 365 days off surfing, please specify number of days (use numbers only)

* 217. How much time off WORK (job or study) did you take due to this TRAUMATIC KNEE injury?

- ○ No time off work
- ○ More than 1 year off work
- ○ Between 1 and 365 days off work, please specify number of days (use numbers only)

Lower Leg & Ankle injury (including feet)

* 218. How many LOWER LEG or ANKLE surfing related injuries have you experienced in the past 12 months?

- 0
- 1
- 2 or more (please specify number of injuries)

Lower Leg & Ankle Injury (including feet)

This questionnaire allows you to report your two worst LOWER LEG or ANKLE surfing related injuries that you experienced in the past 12 months.

* 219. Was the onset of your worst LOWER LEG or ANKLE injury TRAUMATIC or GRADUAL?

- ◯ TRAUMATIC: There was a specific event or sudden impact that occurred while surfing just prior to any symptoms (i.e. pain).
- ◯ GRADUAL: The symptoms (i.e. pain or discomfort) occurred gradually over time. Not one specific event caused this injury, which is aggravated by surfing. This injury may flare up and down, and be affected by the amount of surfing performed.

Second Lower Leg & Ankle Injury (including feet)

* 220. Was the onset of your next worst LOWER LEG or ANKLE injury TRAUMATIC or GRADUAL?

- TRAUMATIC: There was a specific event or sudden impact that occurred while surfing just prior to any symptoms (i.e. pain).
- GRADUAL: The symptoms (i.e. pain or discomfort) occurred gradually over time. Not one specific event caused this injury, which is aggravated by surfing. This injury may flare up and down, and be affected by the amount of surfing performed.

Traumatic Injury (lower leg or ankle including feet)

* 221. Was this TRAUMATIC LOWER LEG or ANKLE injury on your front or back leg

- ◯ Front leg
- ◯ Back leg

* 222. What structure was injured in this TRAUMATIC LOWER LEG or ANKLE injury while surfing in the past 12 months?

- ☐ Skin (e.g. cut, graze, bruise, bite, sting)
- ☐ Bone (e.g. fracture)
- ☐ Joint or ligament (e.g. sprain, cartilage damage)
- ☐ Muscle and tendon (e.g. tear, strain, rupture)
- ☐ Nerve (e.g. compression causing tingling, numbness etc.)
- ☐ Unknown
- ☐ Other (please specify)

* 223. How long did this TRAUMATIC LOWER LEG or ANKLE injury take to recover OR is this injury still persisting?

- ◯ Less than 3 months to recover
- ◯ More than 3 months to recover
- ◯ Still persisting and started less than 3 months ago
- ◯ Still persisting and started more than 3 months ago

* 224. What was the movement or event that occurred just before or contributed to this TRAUMATIC LOWER LEG or ANKLE injury?

- Struck by own board
- Struck by other surfers board
- Striking sea floor
- Striking surface of sea
- Paddling
- Duck diving
- Take off
- Bottom turn
- Top turn
- Cut back
- Re-entry
- Floater
- Aerial
- Riding the face of the wave
- Tube riding
- Encounter with marine life
- Other (please specify)

* 225. What size was the surf when this TRAUMATIC LOWER LEG or ANKLE injury occurred?

- Overhead or greater in height
- Head height or smaller
- Not sure

* 226. What time of year did this TRAUMATIC LOWER LEG or ANKLE injury occur?

- Summer time (December to May)
- Winter time (June to November)

* 227. How did you manage this TRAUMATIC LOWER LEG or ANKLE injury? (you may select more than one option)

- [ ] I had an ACC claim for this injury
- [ ] I went to a healthcare practitioner (e.g. doctor, physiotherapist, osteopath, chiropractor, specialist)
- [ ] I went to a practitioner for traditional Māori healing (e.g. mirimiri, rongoā or karakia)
- [ ] I went to the hospital emergency department
- [ ] I stayed in hospital over night
- [ ] I had surgery
- [ ] I didn't do anything
- [ ] I managed it myself (e.g. ice, pain killers)
- [ ] Other (please specify, e.g. naturopath, homeopath, acupuncture)

* 228. How much time off SURFING did you take due to this TRAUMATIC LOWER LEG or ANKLE injury?

- ○ No time off surfing
- ○ More than 1 year off surfing
- ○ Between 1 and 365 days off surfing, please specify number of days (use numbers only)

* 229. How much time off WORK (job or study) did you take due to this TRAUMATIC LOWER LEG or ANKLE injury?

- ○ No time off work
- ○ More than 1 year off work
- ○ Between 1 and 365 days off work, please specify number of days (use numbers only)

* 230. Have you had another LOWER LEG or ANKLE injury while surfing in the past 12 months?

- ○ Yes
- ○ No

Traumatic Injury (lower leg or ankle including feet 2)

* 231. Was this TRAUMATIC LOWER LEG or ANKLE injury on your front or back leg

- ◯ Front leg
- ◯ Back leg

* 232. What structure was injured in this TRAUMATIC LOWER LEG or ANKLE injury while surfing in the past 12 months?

- ☐ Skin (e.g. cut, graze, bruise, bite, sting)
- ☐ Bone (e.g. fracture)
- ☐ Joint or ligament (e.g. sprain, cartilage damage)
- ☐ Muscle and tendon (e.g. tear, strain, rupture)
- ☐ Nerve (e.g. compression causing tingling, numbness etc.)
- ☐ Unknown
- ☐ Other (please specify)

* 233. How long did this TRAUMATIC LOWER LEG or ANKLE injury take to recover OR is this injury still persisting?

- ◯ Less than 3 months to recover
- ◯ More than 3 months to recover
- ◯ Still persisting and started less than 3 months ago
- ◯ Still persisting and started more than 3 months ago

* 234. What was the movement or event that occurred just before or contributed to this TRAUMATIC LOWER LEG or ANKLE injury?

- ◯ Struck by own board
- ◯ Struck by other surfers board
- ◯ Striking sea floor
- ◯ Striking surface of sea
- ◯ Paddling
- ◯ Duck diving
- ◯ Take off
- ◯ Bottom turn
- ◯ Top turn
- ◯ Cut back
- ◯ Re-entry
- ◯ Floater
- ◯ Aerial
- ◯ Riding the face of the wave
- ◯ Tube riding
- ◯ Encounter with marine life
- ◯ Other (please specify)

* 235. What size was the surf when this TRAUMATIC LOWER LEG or ANKLE injury occurred?

- ◯ Overhead or greater in height
- ◯ Head height or smaller
- ◯ Not sure

* 236. What time of year did this TRAUMATIC LOWER LEG or ANKLE injury occur?

- ◯ Summer time (December to May)
- ◯ Winter time (June to November)

* 237. How did you manage this TRAUMATIC LOWER LEG or ANKLE injury? (you may select more than one option)

- [ ] I had an ACC claim for this injury
- [ ] I went to a healthcare practitioner (e.g. doctor, physiotherapist, osteopath, chiropractor, specialist)
- [ ] I went to a practitioner for traditional Māori healing (e.g. mirimiri, rongoā or karakia)
- [ ] I went to the hospital emergency department
- [ ] I stayed in hospital over night
- [ ] I had surgery
- [ ] I didn't do anything
- [ ] I managed it myself (e.g. ice, pain killers)
- [ ] Other (please specify, e.g. naturopath, homeopath, acupuncture)

* 238. How much time off SURFING did you take due to this TRAUMATIC LOWER LEG or ANKLE injury?

- ○ No time off surfing
- ○ More than 1 year off surfing
- ○ Between 1 and 365 days off surfing, please specify number of days (use numbers only)

* 239. How much time off WORK (job or study) did you take due to this TRAUMATIC LOWER LEG or ANKLE injury?

- ○ No time off work
- ○ More than 1 year off work
- ○ Between 1 and 365 days off work, please specify number of days (use numbers only)

Gradual Injury (head & face)

* 240. What structure was injured in this GRADUAL HEAD or FACE injury while surfing in the past 12 months?

- ☐ Skin (e.g. sea ulcer)
- ☐ Ear (e.g. swimmer's or surfer's ear)
- ☐ Eye (e.g. surfer's eye - pterygium)
- ☐ Unknown
- ☐ Other (please specify)

* 241. How long did this GRADUAL HEAD or FACE injury take to recover OR is this injury still persisting? (may include periods where this injury flares up and down)

- ◯ Less than 3 months to recover
- ◯ More than 3 months to recover
- ◯ Still persisting and started less than 3 months ago
- ◯ Still persisting and started more than 3 months ago

* 242. What causes or aggravates this GRADUAL HEAD or FACE injury? (you may select more than one option)

- ☐ Prolonged sun exposure
- ☐ Prolonged water exposure
- ☐ Unknown
- ☐ Other (please specify)

* 243. How did you manage this GRADUAL HEAD or FACE injury? (you may select more than one option)

- [ ] I went to a health care practitioner (e.g. doctor, physiotherapist, osteopath, chiropractor, specialist)
- [ ] I went to a practitioner for traditional Māori healing (e.g. mirimiri, rongoā or karakia)
- [ ] I stayed in hospital over night
- [ ] I had surgery
- [ ] I didn't do anything
- [ ] I managed it myself (e.g. ice, pain killers)
- [ ] Other (please specify, e.g. naturopath, homeopath, acupuncture)

* 244. How much time off SURFING did you take due to this GRADUAL HEAD or FACE injury

- ○ No time off surfing
- ○ More than 1 year off surfing
- ○ Between 1 and 365 days off surfing, please specify number of days (use numbers only)

* 245. How much time off WORK (job or study) did you take due to this GRADUAL HEAD or FACE injury?

- ○ No time off work
- ○ More than 1 year off work
- ○ Between 1 and 365 days off work, please specify number of days (use numbers only)

* 246. Have you had another HEAD or FACE injury while surfing in the past 12 months?

- ○ Yes
- ○ No

Gradual Injury (head & face 2)

* 247. What structure was injured in this GRADUAL HEAD or FACE injury while surfing in the past 12 months?

- [ ] Skin (e.g. sea ulcer)
- [ ] Ear (e.g. swimmer's or surfer's ear)
- [ ] Eye (e.g. surfer's eye - pterygium)
- [ ] Unknown
- [ ] Other (please specify)

* 248. How long did this GRADUAL HEAD or FACE injury take to recover OR is this injury still persisting? (may include periods where this injury flares up and down)

- ○ Less than 3 months to recover
- ○ More than 3 months to recover
- ○ Still persisting and started less than 3 months ago
- ○ Still persisting and started more than 3 months ago

* 249. What causes or aggravates this GRADUAL HEAD or FACE injury? (you may select more than one option)

- [ ] Prolonged sun exposure
- [ ] Prolonged water exposure
- [ ] Unknown
- [ ] Other (please specify)

* 250. How did you manage this GRADUAL HEAD or FACE injury? (you may select more than one option)

- [ ] I went to a health care practitioner (e.g. doctor, physiotherapist, osteopath, chiropractor, specialist)
- [ ] I went to a practitioner for traditional Māori healing (e.g. mirimiri, rongoā or karakia)
- [ ] I stayed in hospital over night
- [ ] I had surgery
- [ ] I didn't do anything
- [ ] I managed it myself (e.g. ice, pain killers)
- [ ] Other (please specify, e.g. naturopath, homeopath, acupuncture)

* 251. How much time off SURFING did you take due to this GRADUAL HEAD or FACE injury

- ( ) No time off surfing
- ( ) More than 1 year off surfing
- ( ) Between 1 and 365 days off surfing, please specify number of days (use numbers only)

* 252. How much time off WORK (job or study) did you take due to this GRADUAL HEAD or FACE injury?

- ( ) No time off work
- ( ) More than 1 year off work
- ( ) Between 1 and 365 days off work, please specify number of days (use numbers only)

Gradual Injury (neck)

* 253. What structure was injured in this GRADUAL NECK injury while surfing in the past 12 months?

- [ ] Skin (e.g. sea ulcer)
- [ ] Bone (e.g. stress fracture)
- [ ] Joint or ligament (e.g. osteoarthritis, joint degeneration, disc or cartilage damage)
- [ ] Muscle or tendon (e.g. tear, rupture, strain, tendinopathy)
- [ ] Nerve (e.g. compression causing tingling, numbness etc.)
- [ ] Unknown
- [ ] Other (please specify)

* 254. How long did this GRADUAL NECK injury take to recover OR is this injury still persisting? (may include periods where this injury flares up and down)

- ( ) Less than 3 months to recover
- ( ) More than 3 months to recover
- ( ) Still persisting and started less than 3 months ago
- ( ) Still persisting and started more than 3 months ago

* 255. What causes or aggravates this GRADUAL NECK injury? (you may select more than one option)

- [ ] Keeping your head up while paddling
- [ ] Lying on the surfboard
- [ ] Moving your neck while performing turning manoeuvres
- [ ] Unknown
- [ ] Other (please specify)

* 256. How did you manage this GRADUAL NECK injury? (you may select more than one option)

- [ ] I went to a health care practitioner (e.g. doctor, physiotherapist, osteopath, chiropractor, specialist)
- [ ] I went to a practitioner for traditional Māori healing (e.g. mirimiri, rongoā or karakia)
- [ ] I stayed in hospital over night
- [ ] I had surgery
- [ ] I didn't do anything
- [ ] I managed it myself (e.g. ice, pain killers)
- [ ] Other (please specify, e.g. naturopath, homeopath, acupuncture)

* 257. How much time off SURFING did you take due to this GRADUAL NECK injury

- ○ No time off surfing
- ○ More than 1 year off surfing
- ○ Between 1 and 365 days off surfing, please specify number of days (use numbers only)

* 258. How much time off WORK (job or study) did you take due to this GRADUAL NECK injury?

- ○ No time off work
- ○ More than 1 year off work
- ○ Between 1 and 365 days off work, please specify number of days (use numbers only)

* 259. Have you had another NECK injury while surfing in the past 12 months?

- ○ Yes
- ○ No

Gradual Injury (neck 2)

* 260. What structure was injured in this GRADUAL NECK injury while surfing in the past 12 months?

- [ ] Skin (e.g. sea ulcer)
- [ ] Bone (e.g. stress fracture)
- [ ] Joint or ligament (e.g. osteoarthritis, joint degeneration, disc or cartilage damage)
- [ ] Muscle or tendon (e.g. tear, rupture, strain, tendinopathy)
- [ ] Nerve (e.g. compression causing tingling, numbness etc.)
- [ ] Unknown
- [ ] Other (please specify)

* 261. How long did this GRADUAL NECK injury take to recover OR is this injury still persisting? (may include periods where this injury flares up and down)

- ○ Less than 3 months to recover
- ○ More than 3 months to recover
- ○ Still persisting and started less than 3 months ago
- ○ Still persisting and started more than 3 months ago

* 262. What causes or aggravates this GRADUAL NECK injury? (you may select more than one option)

- [ ] Keeping your head up while paddling
- [ ] Lying on the surfboard
- [ ] Moving your neck while performing turning manoeuvres
- [ ] Unknown
- [ ] Other (please specify)

* 263. How did you manage this GRADUAL NECK injury? (you may select more than one option)

- [ ] I went to a health care practitioner (e.g. doctor, physiotherapist, osteopath, chiropractor, specialist)
- [ ] I went to a practitioner for traditional Māori healing (e.g. mirimiri, rongoā or karakia)
- [ ] I stayed in hospital over night
- [ ] I had surgery
- [ ] I didn't do anything
- [ ] I managed it myself (e.g. ice, pain killers)
- [ ] Other (please specify, e.g. naturopath, homeopath, acupuncture)

* 264. How much time off SURFING did you take due to this GRADUAL NECK injury

- ○ No time off surfing
- ○ More than 1 year off surfing
- ○ Between 1 and 365 days off surfing, please specify number of days (use numbers only)

* 265. How much time off WORK (job or study) did you take due to this GRADUAL NECK injury?

- ○ No time off work
- ○ More than 1 year off work
- ○ Between 1 and 365 days off work, please specify number of days (use numbers only)

Gradual Injury (shoulder)

* 266. What structure was injured in this GRADUAL SHOULDER injury while surfing in the past 12 months?

- [ ] Skin (e.g. sea ulcer)
- [ ] Bone (e.g. stress fracture)
- [ ] Joint or ligament (e.g. osteoarthritis, joint degeneration, labrum or cartilage damage)
- [ ] Muscle or tendon (e.g. rotator cuff tear, rupture, strain, tendinopathy)
- [ ] Nerve (e.g. compression causing tingling, numbness etc.)
- [ ] Unknown
- [ ] Other (please specify)

* 267. Have you been given a diagnosis for your GRADUAL SHOULDER injury from a healthcare practitioner (e.g. doctor, physiotherapist, osteopath, specialist)

- ○ Yes
- ○ No

Gradual Injury (shoulder...)

268. What was the diagnosis for your GRADUAL SHOULDER injury?

- [ ] Osteoarthritis/degenerative joint damage
- [ ] Labrum damage
- [ ] Cartilage damage
- [ ] Rotator cuff injury
- [ ] Bursitis
- [ ] Unknown
- [ ] Other (please specify)

* 269. How long did this GRADUAL SHOULDER injury take to recover OR is this injury still persisting? (may include periods where this injury flares up and down)

- ○ Less than 3 months to recover
- ○ More than 3 months to recover
- ○ Still persisting and started less than 3 months ago
- ○ Still persisting and started more than 3 months ago

* 270. What causes or aggravates this GRADUAL SHOULDER injury? (you may select more than one option)

- [ ] Prolonged paddling
- [ ] High intensity paddling into wave
- [ ] Duck diving
- [ ] Pushing down on surfboard to stand up
- [ ] Unknown
- [ ] Other (please specify)

* 271. How did you manage this GRADUAL SHOULDER injury? (you may select more than one option)

- [ ] I went to a health care practitioner (e.g. doctor, physiotherapist, osteopath, chiropractor, specialist)
- [ ] I went to a practitioner for traditional Māori healing (e.g. mirimiri, rongoā or karakia)
- [ ] I stayed in hospital over night
- [ ] I had surgery
- [ ] I didn't do anything
- [ ] I managed it myself (e.g. ice, pain killers)
- [ ] Other (please specify, e.g. naturopath, homeopath, acupuncture)

* 272. How much time off SURFING did you take due to this GRADUAL SHOULDER injury

- ( ) No time off surfing
- ( ) More than 1 year off surfing
- ( ) Between 1 and 365 days off surfing, please specify number of days (use numbers only)

* 273. How much time off WORK (job or study) did you take due to this GRADUAL SHOULDER injury?

- ( ) No time off work
- ( ) More than 1 year off work
- ( ) Between 1 and 365 days off work, please specify number of days (use numbers only)

* 274. Have you had another SHOULDER injury while surfing in the past 12 months?

- ( ) Yes
- ( ) No

Gradual Injury (shoulder 2)

* 275. What structure was injured in this GRADUAL SHOULDER injury while surfing in the past 12 months?

- [ ] Skin (e.g. sea ulcer)
- [ ] Bone (e.g. stress fracture)
- [ ] Joint or ligament (e.g. osteoarthritis, joint degeneration, labrum or cartilage damage)
- [ ] Muscle or tendon (e.g. rotator cuff tear, rupture, strain, tendinopathy)
- [ ] Nerve (e.g. compression causing tingling, numbness etc.)
- [ ] Unknown
- [ ] Other (please specify)

* 276. Have you been given a diagnosis for your GRADUAL SHOULDER injury from a healthcare practitioner (e.g. doctor, physiotherapist, osteopath, specialist)

- ○ Yes
- ○ No

Gradual Injury (shoulder ... 2)

277. What was the diagnosis for your GRADUAL SHOULDER injury?

- [ ] Osteoarthritis/degenerative joint damage
- [ ] Labrum damage
- [ ] Cartilage damage
- [ ] Rotator cuff injury
- [ ] Bursitis
- [ ] Unknown
- [ ] Other (please specify)

* 278. How long did this GRADUAL SHOULDER injury take to recover OR is this injury still persisting? (may include periods where this injury flares up and down)

- ○ Less than 3 months to recover
- ○ More than 3 months to recover
- ○ Still persisting and started less than 3 months ago
- ○ Still persisting and started more than 3 months ago

* 279. What causes or aggravates this GRADUAL SHOULDER injury? (you may select more than one option)

- [ ] Prolonged paddling
- [ ] High intensity paddling into wave
- [ ] Duck diving
- [ ] Pushing down on surfboard to stand up
- [ ] Unknown
- [ ] Other (please specify)

* 280. How did you manage this GRADUAL SHOULDER injury? (you may select more than one option)

- [ ] I went to a health care practitioner (e.g. doctor, physiotherapist, osteopath, chiropractor, specialist)
- [ ] I went to a practitioner for traditional Māori healing (e.g. mirimiri, rongoā or karakia)
- [ ] I stayed in hospital over night
- [ ] I had surgery
- [ ] I didn't do anything
- [ ] I managed it myself (e.g. ice, pain killers)
- [ ] Other (please specify, e.g. naturopath, homeopath, acupuncture)

* 281. How much time off SURFING did you take due to this GRADUAL SHOULDER injury

- ○ No time off surfing
- ○ More than 1 year off surfing
- ○ Between 1 and 365 days off surfing, please specify number of days (use numbers only)

* 282. How much time off WORK (job or study) did you take due to this GRADUAL SHOULDER injury?

- ○ No time off work
- ○ More than 1 year off work
- ○ Between 1 and 365 days off work, please specify number of days (use numbers only)

Gradual Injury (upper back)

* 283. What structure was injured in this GRADUAL UPPER BACK injury while surfing in the past 12 months?

- [ ] Skin (e.g. sea ulcer)
- [ ] Bone (e.g. stress fracture)
- [ ] Joint or ligament (e.g. osteoarthritis, joint degeneration, disc or cartilage damage)
- [ ] Muscle or tendon (e.g. tear, strain, tendinopathy)
- [ ] Nerve (e.g. compression causing tingling, numbness etc.)
- [ ] Unknown
- [ ] Other (please specify)

* 284. How long did this GRADUAL UPPER BACK injury take to recover OR is this injury still persisting? (may include periods where this injury flares up and down)

- ◯ Less than 3 months to recover
- ◯ More than 3 months to recover
- ◯ Still persisting and started less than 3 months ago
- ◯ Still persisting and started more than 3 months ago

* 285. What causes or aggravates this GRADUAL UPPER BACK injury? (you may select more than one option)

- [ ] Prolonged paddling
- [ ] Prolonged lying on surfboard
- [ ] High intensity paddling into wave
- [ ] Performing turning manoeuvres
- [ ] Unknown
- [ ] Other (please specify)

* 286. How did you manage this GRADUAL UPPER BACK injury? (you may select more than one option)

- [ ] I went to a health care practitioner (e.g. doctor, physiotherapist, osteopath, chiropractor, specialist)
- [ ] I went to a practitioner for traditional Māori healing (e.g. mirimiri, rongoā or karakia)
- [ ] I stayed in hospital over night
- [ ] I had surgery
- [ ] I didn't do anything
- [ ] I managed it myself (e.g. ice, pain killers)
- [ ] Other (please specify, e.g. naturopath, homeopath, acupuncture)

* 287. How much time off SURFING did you take due to this GRADUAL UPPER BACK injury

- ○ No time off surfing
- ○ More than 1 year off surfing
- ○ Between 1 and 365 days off surfing, please specify number of days (use numbers only)

* 288. How much time off WORK (job or study) did you take due to this GRADUAL UPPER BACK injury?

- ○ No time off work
- ○ More than 1 year off work
- ○ Between 1 and 365 days off work, please specify number of days (use numbers only)

* 289. Have you had another UPPER BACK injury while surfing in the past 12 months?

- ○ Yes
- ○ No

Gradual Injury (upper back 2)

* 290. What structure was injured in this GRADUAL UPPER BACK injury while surfing in the past 12 months?

- [ ] Skin (e.g. sea ulcer)
- [ ] Bone (e.g. stress fracture)
- [ ] Joint or ligament (e.g. osteoarthritis, joint degeneration, disc or cartilage damage)
- [ ] Muscle or tendon (e.g. tear, strain, tendinopathy)
- [ ] Nerve (e.g. compression causing tingling, numbness etc.)
- [ ] Unknown
- [ ] Other (please specify)

* 291. How long did this GRADUAL UPPER BACK injury take to recover OR is this injury still persisting? (may include periods where this injury flares up and down)

- ○ Less than 3 months to recover
- ○ More than 3 months to recover
- ○ Still persisting and started less than 3 months ago
- ○ Still persisting and started more than 3 months ago

* 292. What causes or aggravates this GRADUAL UPPER BACK injury? (you may select more than one option)

- [ ] Prolonged paddling
- [ ] Prolonged lying on surfboard
- [ ] High intensity paddling into wave
- [ ] Performing turning manoeuvres
- [ ] Unknown
- [ ] Other (please specify)

* 293. How did you manage this GRADUAL UPPER BACK injury? (you may select more than one option)

- [ ] I went to a health care practitioner (e.g. doctor, physiotherapist, osteopath, chiropractor, specialist)
- [ ] I went to a practitioner for traditional Māori healing (e.g. mirimiri, rongoā or karakia)
- [ ] I stayed in hospital over night
- [ ] I had surgery
- [ ] I didn't do anything
- [ ] I managed it myself (e.g. ice, pain killers)
- [ ] Other (please specify, e.g. naturopath, homeopath, acupuncture)

* 294. How much time off SURFING did you take due to this GRADUAL UPPER BACK injury

- ○ No time off surfing
- ○ More than 1 year off surfing
- ○ Between 1 and 365 days off surfing, please specify number of days (use numbers only)

* 295. How much time off WORK (job or study) did you take due to this GRADUAL UPPER BACK injury?

- ○ No time off work
- ○ More than 1 year off work
- ○ Between 1 and 365 days off work, please specify number of days (use numbers only)

Gradual Injury (ribs & sternum - chest)

* 296. What structure was injured in this GRADUAL RIB or STERNUM injury while surfing in the past 12 months?

- [ ] Skin (e.g. sea ulcer)
- [ ] Bone (e.g. stress fracture)
- [ ] Joint or ligament (e.g. osteoarthritis, cartilage damage)
- [ ] Muscle or tendon (e.g. tear, strain, tendinopathy)
- [ ] Nerve (e.g. compression causing tingling, numbness etc.)
- [ ] Unknown
- [ ] Other (please specify)

* 297. How long did this GRADUAL RIB or STERNUM injury take to recover OR is this injury still persisting? (may include periods where this injury flares up and down)

- ○ Less than 3 months to recover
- ○ More than 3 months to recover
- ○ Still persisting and started less than 3 months ago
- ○ Still persisting and started more than 3 months ago

* 298. What causes or aggravates this GRADUAL RIB or STERNUM injury? (you may select more than one option)

- [ ] Prolonged paddling
- [ ] Prolonged lying on surfboard
- [ ] High intensity paddling into wave
- [ ] Performing turning manoeuvres
- [ ] Unknown
- [ ] Other (please specify)

* 299. How did you manage this GRADUAL RIB or STERNUM injury? (you may select more than one option)

- [ ] I went to a health care practitioner (e.g. doctor, physiotherapist, osteopath, chiropractor, specialist)
- [ ] I went to a practitioner for traditional Māori healing (e.g. mirimiri, rongoā or karakia)
- [ ] I stayed in hospital over night
- [ ] I had surgery
- [ ] I didn't do anything
- [ ] I managed it myself (e.g. ice, pain killers)
- [ ] Other (please specify, e.g. naturopath, homeopath, acupuncture)

* 300. How much time off SURFING did you take due to this GRADUAL RIB or STERNUM injury

- No time off surfing
- More than 1 year off surfing
- Between 1 and 365 days off surfing, please specify number of days (use numbers only)

* 301. How much time off WORK (job or study) did you take due to this GRADUAL RIB or STERNUM injury?

- No time off work
- More than 1 year off work
- Between 1 and 365 days off work, please specify number of days (use numbers only)

* 302. Have you had another RIB or STERNUM (chest) injury while surfing in the past 12 months?

- Yes
- No

Gradual Injury (ribs & sternum - chest 2)

* 303. What structure was injured in this GRADUAL RIB or STERNUM injury while surfing in the past 12 months?

- [ ] Skin (e.g. sea ulcer)
- [ ] Bone (e.g. stress fracture)
- [ ] Joint or ligament (e.g. osteoarthritis, cartilage damage)
- [ ] Muscle or tendon (e.g. tear, rupture, strain, tendinopathy)
- [ ] Nerve (e.g. compression causing tingling, numbness etc.)
- [ ] Unknown
- [ ] Other (please specify)

* 304. How long did this GRADUAL RIB or STERNUM injury take to recover OR is this injury still persisting? (may include periods where this injury flares up and down)

- ○ Less than 3 months to recover
- ○ More than 3 months to recover
- ○ Still persisting and started less than 3 months ago
- ○ Still persisting and started more than 3 months ago

* 305. What causes or aggravates this GRADUAL RIB or STERNUM injury? (you may select more than one option)

- [ ] Prolonged paddling
- [ ] Prolonged lying on surfboard
- [ ] High intensity paddling into wave
- [ ] Performing turning manoeuvres
- [ ] Unknown
- [ ] Other (please specify)

* 306. How did you manage this GRADUAL RIB or STERNUM injury? (you may select more than one option)

- [ ] I went to a health care practitioner (e.g. doctor, physiotherapist, osteopath, chiropractor, specialist)
- [ ] I went to a practitioner for traditional Māori healing (e.g. mirimiri, rongoā or karakia)
- [ ] I stayed in hospital over night
- [ ] I had surgery
- [ ] I didn't do anything
- [ ] I managed it myself (e.g. ice, pain killers)
- [ ] Other (please specify, e.g. naturopath, homeopath, acupuncture)

* 307. How much time off SURFING did you take due to this GRADUAL RIB or STERNUM injury

- No time off surfing
- More than 1 year off surfing
- Between 1 and 365 days off surfing, please specify number of days (use numbers only)

* 308. How much time off WORK (job or study) did you take due to this GRADUAL RIB or STERNUM injury?

- No time off work
- More than 1 year off work
- Between 1 and 365 days off work, please specify number of days (use numbers only)

Gradual Injury (arm including elbow, wrist & hand)

* 309. What structure was injured in this GRADUAL ARM (elbow, wrist & hand) injury while surfing in the past 12 months?

- [ ] Skin (e.g. sea ulcer)
- [ ] Bone (e.g. stress fracture)
- [ ] Joint or ligament (e.g. osteoarthritis, cartilage damage)
- [ ] Muscle or tendon (e.g. tear, strain, tendinopathy, golfer's or tennis elbow)
- [ ] Nerve (e.g. carpal tunnel syndrome, other compression causing tingling, numbness etc.)
- [ ] Unknown
- [ ] Other (please specify)

* 310. How long did this GRADUAL ARM injury take to recover OR is this injury still persisting? (may include periods where this injury flares up and down)

- ○ Less than 3 months to recover
- ○ More than 3 months to recover
- ○ Still persisting and started less than 3 months ago
- ○ Still persisting and started more than 3 months ago

* 311. What causes or aggravates this GRADUAL ARM injury? (you may select more than one option)

- [ ] Prolonged paddling
- [ ] High intensity paddling into wave
- [ ] Duck diving
- [ ] Pushing down on surfboard to stand up
- [ ] Unknown
- [ ] Other (please specify)

* 312. How did you manage this GRADUAL ARM injury? (you may select more than one option)

- [ ] I went to a health care practitioner (e.g. doctor, physiotherapist, osteopath, chiropractor, specialist)
- [ ] I went to a practitioner for traditional Māori healing (e.g. mirimiri, rongoā or karakia)
- [ ] I stayed in hospital over night
- [ ] I had surgery
- [ ] I didn't do anything
- [ ] I managed it myself (e.g. ice, pain killers)
- [ ] Other (please specify, e.g. naturopath, homeopath, acupuncture)

* 313. How much time off SURFING did you take due to this GRADUAL ARM injury

- ○ No time off surfing
- ○ More than 1 year off surfing
- ○ Between 1 and 365 days off surfing, please specify number of days (use numbers only)

* 314. How much time off WORK (job or study) did you take due to this GRADUAL ARM injury?

- ○ No time off work
- ○ More than 1 year off work
- ○ Between 1 and 365 days off work, please specify number of days (use numbers only)

* 315. Have you had another ARM (elbow, wrist and hand) injury while surfing in the past 12 months?

- ○ Yes
- ○ No

Gradual Injury (arm including elbow, wrist & hand 2)

* 316. What structure was injured in this GRADUAL ARM (elbow, wrist & hand) injury while surfing in the past 12 months?

- [ ] Skin (e.g. sea ulcer)
- [ ] Bone (e.g. stress fracture)
- [ ] Joint or ligament (e.g. osteoarthritis, cartilage damage)
- [ ] Muscle or tendon (e.g. tear, strain, tendinopathy, golfer's or tennis elbow)
- [ ] Nerve (e.g. carpal tunnel syndrome, other compression causing tingling, numbness etc.)
- [ ] Unknown
- [ ] Other (please specify)

* 317. How long did this GRADUAL ARM injury take to recover OR is this injury still persisting? (may include periods where this injury flares up and down)

- ○ Less than 3 months to recover
- ○ More than 3 months to recover
- ○ Still persisting and started less than 3 months ago
- ○ Still persisting and started more than 3 months ago

* 318. What causes or aggravates this GRADUAL ARM injury? (you may select more than one option)

- [ ] Prolonged paddling
- [ ] High intensity paddling into wave
- [ ] Duck diving
- [ ] Pushing down on surfboard to stand up
- [ ] Unknown
- [ ] Other (please specify)

* 319. How did you manage this GRADUAL ARM injury? (you may select more than one option)

- [ ] I went to a health care practitioner (e.g. doctor, physiotherapist, osteopath, chiropractor, specialist)
- [ ] I went to a practitioner for traditional Māori healing (e.g. mirimiri, rongoā or karakia)
- [ ] I stayed in hospital over night
- [ ] I had surgery
- [ ] I didn't do anything
- [ ] I managed it myself (e.g. ice, pain killers)
- [ ] Other (please specify, e.g. naturopath, homeopath, acupuncture)

* 320. How much time off SURFING did you take due to this GRADUAL ARM injury

- ○ No time off surfing
- ○ More than 1 year off surfing
- ○ Between 1 and 365 days off surfing, please specify number of days (use numbers only)

* 321. How much time off WORK (job or study) did you take due to this GRADUAL ARM injury?

- ○ No time off work
- ○ More than 1 year off work
- ○ Between 1 and 365 days off work, please specify number of days (use numbers only)

Gradual Injury (lower back)

* 322. What structure was injured in this GRADUAL LOWER BACK injury while surfing in the past 12 months?

- [ ] Skin (e.g. sea ulcer)
- [ ] Bone (e.g. stress fracture)
- [ ] Joint or ligament (e.g. osteoarthritis, joint degeneration, disc or cartilage damage)
- [ ] Muscle or tendon (e.g. tear, strain, tendinopathy)
- [ ] Nerve (e.g. sciatica, compression causing tingling, numbness etc.)
- [ ] Unknown
- [ ] Other (please specify)

* 323. How long did this GRADUAL LOWER BACK injury take to recover OR is this injury still persisting? (may include periods where this injury flares up and down)

- ○ Less than 3 months to recover
- ○ More than 3 months to recover
- ○ Still persisting and started less than 3 months ago
- ○ Still persisting and started more than 3 months ago

* 324. What causes or aggravates this GRADUAL LOWER BACK injury? (you may select more than one option)

- [ ] Prolonged paddling
- [ ] Prolonged lying on surfboard
- [ ] Prolonged sitting on board
- [ ] Standing up phase
- [ ] Performing turning manoeuvres
- [ ] Landing aerials
- [ ] Unknown
- [ ] Other (please specify)

* 325. How did you manage this GRADUAL LOWER BACK injury? (you may select more than one option)

- [ ] I went to a health care practitioner (e.g. doctor, physiotherapist, osteopath, chiropractor, specialist)
- [ ] I went to a practitioner for traditional Māori healing (e.g. mirimiri, rongoā or karakia)
- [ ] I stayed in hospital over night
- [ ] I had surgery
- [ ] I didn't do anything
- [ ] I managed it myself (e.g. ice, pain killers)
- [ ] Other (please specify, e.g. naturopath, homeopath, acupuncture)

* 326. How much time off SURFING did you take due to this GRADUAL LOWER BACK injury

- ○ No time off surfing
- ○ More than 1 year off surfing
- ○ Between 1 and 365 days off surfing, please specify number of days (use numbers only)

* 327. How much time off WORK (job or study) did you take due to this GRADUAL LOWER BACK injury?

- ○ No time off work
- ○ More than 1 year off work
- ○ Between 1 and 365 days off work, please specify number of days (use numbers only)

* 328. Have you had another LOWER BACK injury while surfing in the past 12 months?

- ○ Yes
- ○ No

Gradual Injury (lower back 2)

* 329. What structure was injured in this GRADUAL LOWER BACK injury while surfing in the past 12 months?

- [ ] Skin (e.g. sea ulcer)
- [ ] Bone (e.g. stress fracture)
- [ ] Joint or ligament (e.g. osteoarthritis, joint degeneration, disc or cartilage damage)
- [ ] Muscle or tendon (e.g. tear, strain, tendinopathy)
- [ ] Nerve (e.g. sciatica, compression causing tingling, numbness etc.)
- [ ] Unknown
- [ ] Other (please specify)

* 330. How long did this GRADUAL LOWER BACK injury take to recover OR is this injury still persisting? (may include periods where this injury flares up and down)

- ○ Less than 3 months to recover
- ○ More than 3 months to recover
- ○ Still persisting and started less than 3 months ago
- ○ Still persisting and started more than 3 months ago

* 331. What causes or aggravates this GRADUAL LOWER BACK injury? (you may select more than one option)

- [ ] Prolonged paddling
- [ ] Prolonged lying on surfboard
- [ ] Prolonged sitting on board
- [ ] Standing up phase
- [ ] Performing turning manoeuvres
- [ ] Landing aerials
- [ ] Unknown
- [ ] Other (please specify)

* 332. How did you manage this GRADUAL LOWER BACK injury? (you may select more than one option)

- [ ] I went to a health care practitioner (e.g. doctor, physiotherapist, osteopath, chiropractor, specialist)
- [ ] I went to a practitioner for traditional Māori healing (e.g. mirimiri, rongoā or karakia)
- [ ] I stayed in hospital over night
- [ ] I had surgery
- [ ] I didn't do anything
- [ ] I managed it myself (e.g. ice, pain killers)
- [ ] Other (please specify, e.g. naturopath, homeopath, acupuncture)

* 333. How much time off SURFING did you take due to this GRADUAL LOWER BACK injury

- No time off surfing
- More than 1 year off surfing
- Between 1 and 365 days off surfing, please specify number of days (use numbers only)

* 334. How much time off WORK (job or study) did you take due to this GRADUAL LOWER BACK injury?

- No time off work
- More than 1 year off work
- Between 1 and 365 days off work, please specify number of days (use numbers only)

Gradual Injury (hip or groin)

* 335. Was this GRADUAL HIP or GROIN injury on your front or back leg

- ◯ Front leg
- ◯ Back leg

* 336. What structure was injured in this GRADUAL HIP or GROIN injury while surfing in the past 12 months?

- ☐ Skin (e.g. sea ulcer)
- ☐ Bone (e.g. stress fracture)
- ☐ Joint or ligament (e.g. osteoarthritis, joint degeneration, labrum or cartilage damage)
- ☐ Muscle or tendon (e.g. tear, strain, tendinopathy)
- ☐ Nerve (e.g. compression causing tingling, numbness etc.)
- ☐ Unknown
- ☐ Other (please specify)

* 337. How long did this GRADUAL HIP or GROIN injury take to recover OR is this injury still persisting? (may include periods where this injury flares up and down)

- ◯ Less than 3 months to recover
- ◯ More than 3 months to recover
- ◯ Still persisting and started less than 3 months ago
- ◯ Still persisting and started more than 3 months ago

* 338. What causes or aggravates this GRADUAL HIP or GROIN injury? (you may select more than one option)

- [ ] Prolonged sitting on surfboard
- [ ] Stand up phase
- [ ] Duck diving
- [ ] Performing turning manoeuvres
- [ ] Tube riding
- [ ] Landing aerials
- [ ] Unknown
- [ ] Other (please specify)

* 339. How did you manage this GRADUAL HIP or GROIN injury? (you may select more than one option)

- [ ] I went to a health care practitioner (e.g. doctor, physiotherapist, osteopath, chiropractor, specialist)
- [ ] I went to a practitioner for traditional Māori healing (e.g. mirimiri, rongoā or karakia)
- [ ] I stayed in hospital over night
- [ ] I had surgery
- [ ] I didn't do anything
- [ ] I managed it myself (e.g. ice, pain killers)
- [ ] Other (please specify, e.g. naturopath, homeopath, acupuncture)

* 340. How much time off SURFING did you take due to this GRADUAL HIP or GROIN injury

- ( ) No time off surfing
- ( ) More than 1 year off surfing
- ( ) Between 1 and 365 days off surfing, please specify number of days (use numbers only)

* 341. How much time off WORK (job or study) did you take due to this GRADUAL HIP or GROIN injury?

- No time off work
- More than 1 year off work
- Between 1 and 365 days off work, please specify number of days (use numbers only)

* 342. Have you had another HIP or GROIN injury while surfing in the past 12 months?

- Yes
- No

Gradual Injury (hip or groin 2)

* 343. Was this GRADUAL HIP or GROIN injury on your front or back leg

- Front leg
- Back leg

* 344. What structure was injured in this GRADUAL HIP or GROIN injury while surfing in the past 12 months?

- [ ] Skin (e.g. sea ulcer)
- [ ] Bone (e.g. stress fracture)
- [ ] Joint or ligament (e.g. osteoarthritis, joint degeneration, labrum or cartilage damage)
- [ ] Muscle or tendon (e.g. tear, strain, tendinopathy)
- [ ] Nerve (e.g. compression causing tingling, numbness etc.)
- [ ] Unknown
- [ ] Other (please specify)

* 345. How long did this GRADUAL HIP or GROIN injury take to recover OR is this injury still persisting? (may include periods where this injury flares up and down)

- Less than 3 months to recover
- More than 3 months to recover
- Still persisting and started less than 3 months ago
- Still persisting and started more than 3 months ago

* 346. What causes or aggravates this GRADUAL HIP or GROIN injury? (you may select more than one option)

- [ ] Prolonged sitting on surfboard
- [ ] Stand up phase
- [ ] Duck diving
- [ ] Performing turning manoeuvres
- [ ] Tube riding
- [ ] Landing aerials
- [ ] Unknown
- [ ] Other (please specify)

* 347. How did you manage this GRADUAL HIP or GROIN injury? (you may select more than one option)

- [ ] I went to a health care practitioner (e.g. doctor, physiotherapist, osteopath, chiropractor, specialist)
- [ ] I went to a practitioner for traditional Māori healing (e.g. mirimiri, rongoā or karakia)
- [ ] I stayed in hospital over night
- [ ] I had surgery
- [ ] I didn't do anything
- [ ] I managed it myself (e.g. ice, pain killers)
- [ ] Other (please specify, e.g. naturopath, homeopath, acupuncture)

* 348. How much time off SURFING did you take due to this GRADUAL HIP or GROIN injury

- ○ No time off surfing
- ○ More than 1 year off surfing
- ○ Between 1 and 365 days off surfing, please specify number of days (use numbers only)

* 349. How much time off WORK (job or study) did you take due to this GRADUAL HIP or GROIN injury?

- ◯ No time off work
- ◯ More than 1 year off work
- ◯ Between 1 and 365 days off work, please specify number of days (use numbers only)

[ ]

Gradual Injury (knee)

* 350. Was this GRADUAL KNEE injury on your front or back leg

- ◯ Front leg
- ◯ Back leg

* 351. What structure was injured in this GRADUAL KNEE injury while surfing in the past 12 months?

- ☐ Skin (e.g. sea ulcer)
- ☐ Bone (e.g. stress fracture)
- ☐ Joint or ligament (e.g. osteoarthritis, ligament sprain, cartilage/meniscus damage, patellofemoral syndrome)
- ☐ Muscle or tendon (e.g. tear, strain, tendinopathy)
- ☐ Nerve (e.g. compression causing tingling, numbness etc.)
- ☐ Unknown
- ☐ Other (please specify)

* 352. How long did this GRADUAL KNEE injury take to recover OR is this injury still persisting? (may include periods where this injury flares up and down)

- ◯ Less than 3 months to recover
- ◯ More than 3 months to recover
- ◯ Still persisting and started less than 3 months ago
- ◯ Still persisting and started more than 3 months ago

* 353. What causes or aggravates this GRADUAL KNEE injury? (you may select more than one option)

- [ ] Stand up phase
- [ ] Duck diving
- [ ] Performing turning manoeuvres
- [ ] Tube riding
- [ ] Landing aerials
- [ ] Unknown
- [ ] Other (please specify)

* 354. How did you manage this GRADUAL KNEE injury? (you may select more than one option)

- [ ] I went to a health care practitioner (e.g. doctor, physiotherapist, osteopath, chiropractor, specialist)
- [ ] I went to a practitioner for traditional Māori healing (e.g. mirimiri, rongoā or karakia)
- [ ] I stayed in hospital over night
- [ ] I had surgery
- [ ] I didn't do anything
- [ ] I managed it myself (e.g. ice, pain killers)
- [ ] Other (please specify, e.g. naturopath, homeopath, acupuncture)

* 355. How much time off SURFING did you take due to this GRADUAL KNEE injury

- ( ) No time off surfing
- ( ) More than 1 year off surfing
- ( ) Between 1 and 365 days off surfing, please specify number of days (use numbers only)

* 356. How much time off WORK (job or study) did you take due to this GRADUAL KNEE injury?

- ( ) No time off work
- ( ) More than 1 year off work
- ( ) Between 1 and 365 days off work, please specify number of days (use numbers only)

* 357. Have you had another KNEE injury while surfing in the past 12 months?

- ◯ Yes
- ◯ No

Gradual Injury (knee 2)

* 358. Was this GRADUAL KNEE injury on your front or back leg

- ◯ Front leg
- ◯ Back leg

* 359. What structure was injured in this GRADUAL KNEE injury while surfing in the past 12 months?

- ☐ Skin (e.g. sea ulcer)
- ☐ Bone (e.g. stress fracture)
- ☐ Joint or ligament (e.g. osteoarthritis, ligament sprain, cartilage/meniscus damage, patellofemoral syndrome)
- ☐ Muscle or tendon (e.g. tear, strain, tendinopathy)
- ☐ Nerve (e.g. compression causing tingling, numbness etc.)
- ☐ Unknown
- ☐ Other (please specify)

* 360. How long did this GRADUAL KNEE injury take to recover OR is this injury still persisting? (may include periods where this injury flares up and down)

- ◯ Less than 3 months to recover
- ◯ More than 3 months to recover
- ◯ Still persisting and started less than 3 months ago
- ◯ Still persisting and started more than 3 months ago

* 361. What causes or aggravates this GRADUAL KNEE injury? (you may select more than one option)

- [ ] Stand up phase
- [ ] Duck diving
- [ ] Performing turning manoeuvres
- [ ] Tube riding
- [ ] Landing aerials
- [ ] Unknown
- [ ] Other (please specify)

* 362. How did you manage this GRADUAL KNEE injury? (you may select more than one option)

- [ ] I went to a health care practitioner (e.g. doctor, physiotherapist, osteopath, chiropractor, specialist)
- [ ] I went to a practitioner for traditional Māori healing (e.g. mirimiri, rongoā or karakia)
- [ ] I stayed in hospital over night
- [ ] I had surgery
- [ ] I didn't do anything
- [ ] I managed it myself (e.g. ice, pain killers)
- [ ] Other (please specify, e.g. naturopath, homeopath, acupuncture)

* 363. How much time off SURFING did you take due to this GRADUAL KNEE injury

- ○ No time off surfing
- ○ More than 1 year off surfing
- ○ Between 1 and 365 days off surfing, please specify number of days (use numbers only)

* 364. How much time off WORK (job or study) did you take due to this GRADUAL KNEE injury?

- ○ No time off work
- ○ More than 1 year off work
- ○ Between 1 and 365 days off work, please specify number of days (use numbers only)

Gradual Injury (lower leg & ankle including feet)

* 365. Was this GRADUAL LOWER LEG or ANKLE injury on your front or back leg

- ◯ Front leg
- ◯ Back leg

* 366. What structure was injured in this GRADUAL LOWER LEG or ANKLE injury while surfing in the past 12 months?

- ☐ Skin (e.g. sea ulcer)
- ☐ Bone (e.g. stress fracture)
- ☐ Joint or ligament (e.g. osteoarthritis, ligament sprain, cartilage damage)
- ☐ Muscle or tendon (e.g. tear, strain, achilles tendinopathy)
- ☐ Nerve (e.g. compression causing tingling, numbness etc.)
- ☐ Unknown
- ☐ Other (please specify)

* 367. How long did this GRADUAL LOWER LEG or ANKLE injury take to recover OR is this injury still persisting? (may include periods where this injury flares up and down)

- ◯ Less than 3 months to recover
- ◯ More than 3 months to recover
- ◯ Still persisting and started less than 3 months ago
- ◯ Still persisting and started more than 3 months ago

* 368. What causes or aggravates this GRADUAL LOWER LEG or ANKLE injury? (you may select more than one option)

- [ ] Stand up phase
- [ ] Duck diving
- [ ] Performing turning manoeuvres
- [ ] Tube riding
- [ ] Landing aerials
- [ ] Unknown
- [ ] Other (please specify)

* 369. How did you manage this GRADUAL LOWER LEG or ANKLE injury? (you may select more than one option)

- [ ] I went to a health care practitioner (e.g. doctor, physiotherapist, osteopath, chiropractor, specialist)
- [ ] I went to a practitioner for traditional Māori healing (e.g. mirimiri, rongoā or karakia)
- [ ] I stayed in hospital over night
- [ ] I had surgery
- [ ] I didn't do anything
- [ ] I managed it myself (e.g. ice, pain killers)
- [ ] Other (please specify, e.g. naturopath, homeopath, acupuncture)

* 370. How much time off SURFING did you take due to this GRADUAL LOWER LEG or ANKLE injury

- ( ) No time off surfing
- ( ) More than 1 year off surfing
- ( ) Between 1 and 365 days off surfing, please specify number of days (use numbers only)

* 371. How much time off WORK (job or study) did you take due to this GRADUAL LOWER LEG or ANKLE injury?

- No time off work
- More than 1 year off work
- Between 1 and 365 days off work, please specify number of days (use numbers only)

* 372. Have you had another LOWER LEG or ANKLE injury while surfing in the past 12 months?

- Yes
- No

Gradual Injury (lower leg & ankle including feet 2)

* 373. Was this GRADUAL LOWER LEG or ANKLE injury on your front or back leg

- ◯ Front leg
- ◯ Back leg

* 374. What structure was injured in this GRADUAL LOWER LEG or ANKLE injury while surfing in the past 12 months?

- ☐ Skin (e.g. sea ulcer)
- ☐ Bone (e.g. stress fracture)
- ☐ Joint or ligament (e.g. osteoarthritis, ligament sprain, cartilage damage)
- ☐ Muscle or tendon (e.g. tear, strain, achilles tendinopathy)
- ☐ Nerve (e.g. compression causing tingling, numbness etc.)
- ☐ Unknown
- ☐ Other (please specify)

* 375. How long did this GRADUAL LOWER LEG or ANKLE injury take to recover OR is this injury still persisting? (may include periods where this injury flares up and down)

- ◯ Less than 3 months to recover
- ◯ More than 3 months to recover
- ◯ Still persisting and started less than 3 months ago
- ◯ Still persisting and started more than 3 months ago

* 376. What causes or aggravates this GRADUAL LOWER LEG or ANKLE injury? (you may select more than one option)

- [ ] Stand up phase
- [ ] Duck diving
- [ ] Performing turning manoeuvres
- [ ] Tube riding
- [ ] Landing aerials
- [ ] Unknown
- [ ] Other (please specify)

* 377. How did you manage this GRADUAL LOWER LEG or ANKLE injury? (you may select more than one option)

- [ ] I went to a health care practitioner (e.g. doctor, physiotherapist, osteopath, chiropractor, specialist)
- [ ] I went to a practitioner for traditional Māori healing (e.g. mirimiri, rongoā or karakia)
- [ ] I stayed in hospital over night
- [ ] I had surgery
- [ ] I didn't do anything
- [ ] I managed it myself (e.g. ice, pain killers)
- [ ] Other (please specify, e.g. naturopath, homeopath, acupuncture)

* 378. How much time off SURFING did you take due to this GRADUAL LOWER LEG or ANKLE injury

- ( ) No time off surfing
- ( ) More than 1 year off surfing
- ( ) Between 1 and 365 days off surfing, please specify number of days (use numbers only)

* 379. How much time off WORK (job or study) did you take due to this GRADUAL LOWER LEG or ANKLE injury?

- No time off work
- More than 1 year off work
- Between 1 and 365 days off work, please specify number of days (use numbers only)

Surfer's Ear - Exostosis

* 380. Have you been given a diagnosis by a doctor of SURFER'S EAR - EXTERNAL AUDITORY CANAL EXOSTOSIS (abnormal bony growth in the ear canal)?

- ◯ No
- ◯ Yes, in the right ear
- ◯ Yes, in the left ear
- ◯ Yes, in both ears

Contact details

Please bear in mind that by providing your contact details your data will be identifiable, but be assured it will be treated with complete confidentiality.

If you wish to remain completely anonymous scroll down and click "next".

381. If you agree for the researchers to contact you regarding information in this questionnaire, for example to clarify any of your responses, select I agree and enter your contact details below.

- ○ I agree to be contacted by the researchers

382. If you are interested in being a potential participant for future research on surfing select I agree and enter your contact details below.

- ○ I agree to be contacted regarding participation in future research

383. The winner of the O'NEILL HYPERFREAK 4/3 WETSUIT has already been drawn.

- ○ ...

384. Please enter your contact details below or leave blank and click "next"

Name: ______

Email Address: ______

Phone Number: ______

Thank you

Thank you for taking the time to complete our questionnaire (Unitec Institute of Technology)

We wish to thank O'NEILL for their generosity and support towards this research. The recipient of the O'NEILL HYPERFREAK 4/3mm WETSUIT will be notified my email or phone by a member of the research team by June 2016. A similar wetsuit will be substituted if there is a female recipient.

Please click "Done" to finish the survey
